# Toxin-Derived Peptides as Potentials Weapons Against Cancer

**DOI:** 10.3390/pharmaceutics18060722

**Published:** 2026-06-11

**Authors:** Bárbara Pinto, Joaquim Teixeira de Avelar Júnior, Edleusa Marques Lima, Lívia Ramos Santiago, Rosy Iara Maciel de Azambuja Ribeiro, Renata Toscano Simões, Cristina Moraes Junta, Rachel Carolina Souza Fagundes, Ana Clara Costa Velozo, Hassan Bousbaa, Miriam Teresa Paz Lopes, Juliana Carvalho-Tavares, Elaine Maria de Souza-Fagundes, Maria Elena de Lima

**Affiliations:** 1 Departamento de Fisiologia e Biofísica, Instituto de Ciências Biológicas, Universidade Federal de Minas Gerais, Avenida Presidente Antônio Carlos, 6627, Belo Horizonte 31270-901, Brazil; rachelcsfgundes@gmail.com (R.C.S.F.); julianact2015@gmail.com (J.C.-T.); elainefagundes@ufmg.br (E.M.d.S.-F.); 2 UNIPRO—Oral Pathology and Rehabilitation Research Unit, University Institute of Health Sciences (IUCS), CESPU, Rua Central de Gandra 1317, 4585-116 Gandra, Portugal; hassan.bousbaa@iucs.cespu.pt; 3 UCIBIO—Applied Molecular Biosciences Unit, Translational Toxicology Research Laboratory (TOXRUN), University Institute of Health Sciences (IUCS), CESPU, Rua Central de Gandra 1317, 4585-116 Gandra, Portugal; 4 Programa de Pós-Graduação em Inovação Tecnológica, Universidade Federal de Minas Gerais, Avenida Presidente Antônio Carlos, 6627, Belo Horizonte 31270-901, Brazil; avelarjt@gmail.com; 5 Núcleo de Ensino Pesquisa e Inovação—NEPI, Pesquisa Clínica Instituto Mário Penna, Rua Gentios, 1420, Terceiro Pavimento, Conjunto Santa Maria, Belo Horizonte 30380-472, Brazil; edleusa_marques@yahoo.com.br; 6 Laboratório de Patologia Experimental, Universidade Federal de São João del-Rei, Rua Sebastião Gonçalves Coelho, 400, Bairro Chanadour, Divinópolis 35501-296, Brazil; santiagoliviar@gmail.com (L.R.S.); rosy@ufsj.edu.br (R.I.M.d.A.R.); 7 Programa de Pós-Graduação em Medicina-Biomedicina, Faculdade de Saúde Santa Casa de Belo Horizonte (PPG/FSCBH), Av. dos Andradas, 2688 Bairro Santa Efigênia, Belo Horizonte 30110-005, Brazil; rtsimoes@faculdadesantacasabh.edu.br (R.T.S.); cristinajunta@faculdadesantacasabh.edu.br (C.M.J.); anaclaracvelozo@outlook.com (A.C.C.V.); 8 Associate Laboratory i4HB—Institute for Health and Bioeconomy, University Institute of Health Sciences (IUCS), CESPU, Rua Central de Gandra 1317, 4585-116 Gandra, Portugal; 9 Laboratório de Substâncias Antitumorais, Departamento Farmacologia, Instituto de Ciências Biológicas, Universidade Federal de Minas Gerais, Avenida Presidente Antônio Carlos, 6627, Belo Horizonte 31270-901, Brazil; mtpl.ufmg@gmail.com

**Keywords:** animal toxins, toxin peptides, anticancer activity, mechanism of action, therapeutic applications of peptides, drug delivery systems, antimicrobial peptides (AMPs)

## Abstract

Cancer, a longstanding global challenge, remains a leading cause of death, prompting an urgent search for effective treatments. Conventional therapies, while prevalent, often cause adverse effects due to their lack of specificity. This review explores an innovative approach, focusing on animal toxins as a rich source of bioactive compounds which have demonstrated efficacy against cancer cells. Peptides from various species, including scorpions, snakes, bees, spiders, and frogs, show promising antiproliferative and cytotoxic effects. Emphasizing the most prevalent types of cancer, this review outlines the discovery and development stages of potential anticancer drugs derived from toxin peptides. The comprehensive overview includes in vitro and in vivo assessments of their anticancer activity and toxicity. This pioneering exploration extends to different tumors, offering valuable insights into mechanisms of action and potential therapeutic applications. The findings highlight a paradigm shift in cancer research, showcasing the potential of toxin-derived compounds for developing targeted and efficient cancer therapies with reduced side effects.

## 1. Introduction

From antiquity to the present day, cancer remains a difficult challenge to overcome, being one of the main causes of death worldwide. This extensive array of diseases can impact different regions of the body, inducing alterations in cellular physiology that result in the unbridled growth of normal cells, infiltration into neighboring tissues, and the potential for dissemination to distant organs, a process known as metastasis [1].

According to the most recent GLOBOCAN estimates, approximately 20 million new cancer cases and 9.7 million cancer-related deaths occurred worldwide in 2022. Lung cancer was the most frequently diagnosed malignancy, accounting for 12.4% of all new cancer cases, followed by female breast cancer (11.6%), colorectal cancer (9.6%), and prostate cancer (7.3%). Lung cancer also remained the leading cause of cancer-related mortality, representing 18.7% of all cancer deaths worldwide, followed by colorectal, liver, female breast, and stomach cancers. Furthermore, demographic projections indicate that the global cancer burden may exceed 35 million new cases by 2050, representing an increase of approximately 77% compared with 2022 estimates [2]. Therapeutic strategies depend on the type of cancer, the degree of how advanced it is, the targets and what treatments are available. Common treatments available in addition to traditional therapy (surgery, chemotherapy, and radiotherapy) include targeted therapy, immunotherapy, stem cell or bone marrow transplant, and hormone therapy. Other adjuvant treatments include dietary supplements, hyperthermia, and the use of lasers, blood transfusion, and transplantation [3]. However, the available treatments, unfortunately, cause adverse effects due to their lack of specificity and toxicity to normal cells, including allergic reactions, immunosuppression, neurotoxicity, hepatotoxicity, nephrotoxicity, ototoxicity, mucositis, and hair loss, among others. Therefore, the search for new drugs is urgent, and many preclinical and clinical trials are under way.

Animal toxins represent a rich source that have been found to contain a wide variety of bioactive compounds, including peptides, proteins, and small molecules, and some of them have demonstrated antiproliferative and cytotoxic effects against cancer cells. These bioactive molecules have shown high specificity and selectivity toward proteins and proteins subtypes that are often overexpressed or mutated in cancer cells, making them valuable tools for developing new cancer drugs. By isolating and characterizing these molecules, researchers can gain insight into their mechanisms of action and potential therapeutic applications [4]. Toxins peptides have shown great potential in inducing cancer cell death and enhancing the efficacy of chemotherapy and radiotherapy. In addition, they have been found to suppress angiogenesis and epithelial-to-mesenchymal transition, which are processes that contribute to cancer progression and metastasis [5].

A survey of the PubMed and Web of Science databases indicates that venoms and secretions from snakes, bees, scorpions, cone snails, spiders, and frogs represent the animal sources most extensively investigated in toxinology. Notably, peptides derived from snake, bee, and scorpion venoms comprise the majority of animal toxin-derived peptides evaluated for anticancer activity. Their peptides with anticancer activity follow the pattern found in each type of venom or poison, with bees, amphibians, and spiders having more alpha helix peptides [6,7,8,9], as well scorpions more commonly with predominant beta sheet conformation [10], even though in scorpions, both conformations (alpha and beta) exist in peptide folding [10].

**Figure 1:**
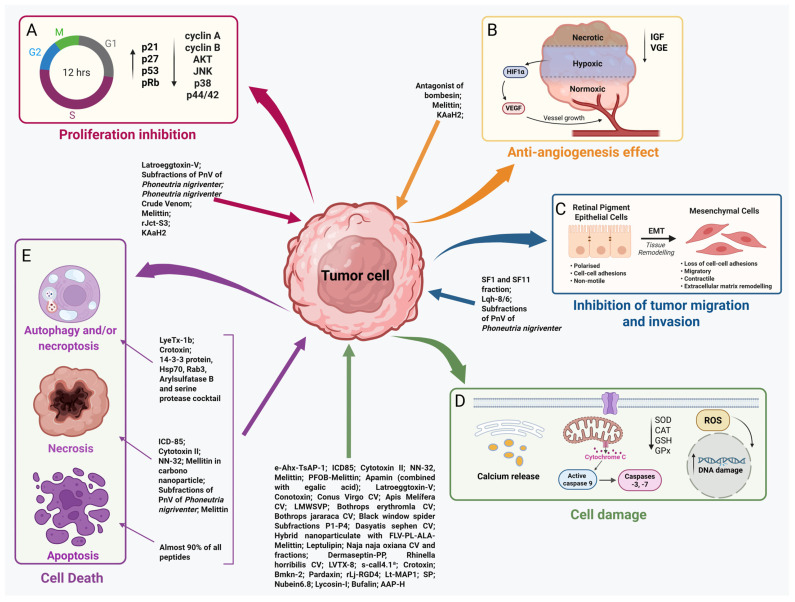
Mechanisms of action from animal toxins/peptides acting on different cancer types. The peptides can predominantly act on various signaling pathways, including the inhibition of cancer cells’ proliferation (**A**); inhibition of angiogenesis (**B**); reduction of tumor migration and invasion (**C**); induction of cellular damage through the activation of caspase cascades, reactive oxygen species, and direct DNA damage (**D**); and through the induction of autophagy, necroptosis, necrosis, and apoptosis (**E**), leading to cancer cell death. Colored arrows indicate the association between the peptides/toxins and their corresponding predominant mechanism of action represented in each panel. Abbreviations: CAT: catalase; EMT: epithelial-to-mesenchymal transition; GPx: glutathione peroxidase; GSH: Glutathione or γ-glutamyl-cysteinyl-glycine; IGF: insulin-like growth factor; ROS: reactive oxygen species; SOD: superoxide dismutase; VEGF: vascular endothelial growth factor. Created in BioRender. Pinto, B. (2026) https://BioRender.com/wm67q9c (accessed on 25 June 2025).

In this review, we present an overview of the discovery and development stages of potential anticancer drugs derived from peptides isolated from toxins of different species, along with their synthetic derivatives and crude toxins. The focus is on exploring their efficacy against different types of cancer. Among animal toxin-derived compounds, peptides obtained from scorpions, snakes, bees, spiders, and frogs have been extensively investigated. The topics are organized based on the type of cancer, with the most significant number of studies involving peptides, some derivatives of them, and crude toxins. Although breast cancer models account for a substantial proportion of the studies investigating toxin-derived peptides, reflecting the extensive interest in this tumor type, other highly prevalent and lethal cancers remain poorly explored. During our literature search, we did not identify studies evaluating toxin-derived peptides against gastric or skin cancers, despite their global clinical relevance. Likewise, pancreatic cancer and neuroblastoma, which are associated with high mortality rates, were scarcely investigated in the context of anticancer peptides derived from animal toxins. Snakes and scorpions had the highest amount of peptides, and it reflects the quantity of total papers studying these animals, according to a PubMed search. The principal anticancer mechanisms associated with the toxin-derived peptides discussed throughout this review are summarized in Figure 1. 

### 1.1. Breast Cancer

Breast cancer stands as the most prevalent form of cancer and is the leading cause of cancer-related fatalities among women worldwide [11]. The majority of toxin-derivated peptides research targeting breast cancer cells therapy involves the use of peptides from snake, scorpion, and bee venoms (Table 1). Most of the in vitro cellular models have used lineages derived from female breast cancer, including the triple-negative resistant cell line MDA-MB-231, and other variants, such as MDA-MB-435 (lacking receptors of estrogen and progesterone) and MCF-7 (estrogen and progesterone receptors positive). As non-tumor cell models, MCF-10 (epithelial lineage from mammary gland) and HEK-293 (a human embryonic kidney cell) are widely used in the biopharmaceutical and research areas.

The venom of scorpions such as *Androctonus australis* and *Tityus serrulatus* are the most studied, and from them, three peptides with activity against breast cancer have been yield. Peptides KAaH1 and KAaH2 from *A. australis* were observed to inhibit triple-negative cancer cells MDA-MB-231 migration at 50 µg/mL. This inhibition appears be dependent of Kv1.1 and Kv1.3 channels, even though a better controlled experiment and ion channel silencing experiments should be performed to test this proposition with more rigor [12]. From *Tityus serrulatus*, the ε-Ahx-TsAP-1 peptide was isolated, which was modified to be linked to acid amino hexanoic. This modified peptide shows activity against MCF-7 half-maximal inhibitory concentration (IC_50_ = 10 μM) and MDA-MB-231 cells (IC_50_ = 20 μM) and induced DNA fragmentation after 24 h of treatment, suggesting apoptosis induction [13]. Another studied scorpion venom peptide, chlorotoxin (CTX), derived from *Leiurus quinquestriatus*, is recognized for its cytotoxic effects against glioma cells and various and different breast cancer lineages at micromolar concentrations (5 μM), including the MCF-7, MDA-MB-231, and T47D cell lines. CTX inhibits MCF-7 breast cancer cell viability, proliferation, and migration by directly binding to estrogen receptor alpha (ERα), a central regulator of tumor growth. This interaction suppresses ERα expression and disrupts the ERα/vasodilator-stimulated phosphoprotein (VASP) signaling pathway, reducing tumor development. These results were confirmed in vivo through inhibition of tumor growth in MCF-7 orthotopic xenografts [14].

Three peptides were found in *Mesobuthus martensii Karsch* (formerly known as *Buthus martensii Karsch*, a Chinese scorpion), BmKn-2, BmK analgesic-antitumor peptide (AGAP), and AGAP linked by N-terminal to fusion peptide ATF, with relevant antiproliferative, pro-apoptotic, and anti-metastatic activities against breast cancer cells. The first one is a short antimicrobial peptide, also active against the CHMp-5b cell line (canine breast cancer). The IC_50_ values at 24 h to CHMp-5b (metastatic cells) and CHMp-13a (non-metastatic cells) were 30 µg/mL and 54 µg/mL, respectively. The BmKn-2 induces apoptosis and overexpression of the proapoptotic protein Bax and suppresses Bcl-2, an antiapoptotic protein, triggering the mitochondrial apoptotic cell death [15]. BmK AGAP is a peptide with anticancer and antinociceptive activities, two important effects in cancer treatment. In vitro studies demonstrated cytotoxicity at IC values of 40 and 50 μM against MCF-7 and MDA-MB-231, respectively. Moreover, the peptide also inhibits the epithelial–mesenchymal transition (EMT) of breast cancer lines by reducing the expression of N-cadherin and increasing E-cadherin, in addition to downregulating PTX3, a protein involved in inflammation and tumor progression, via Nav1.5 suppression. In this way, it inhibits the NF-κB and Wnt/β-catenin signaling pathways, which control cell proliferation, survival and metastasis. In an in vivo assay, BmK AGAP, with a dose of 0.5 or 1.0 mg/kg, inhibits breast tumor growth, with stem-like features and epithelial-to-mesenchymal transition in a BALB/c xenograft MDA-MB-231 model [16]. Moreover, a study using AGAP fused to ATF (the amino-terminal fragment of urokinase-type plasminogen activator (uPA), which contains the urokinase plasminogen activator receptor (uPAR)-binding region but lacks the catalytic domain) demonstrated that ATF enhances the activity of AGAP. In this context, ATF functions as a specific cell-targeting domain, facilitating the delivery of AGAP to the surface of MDA-MB-231 cells. The resulting fusion protein, termed ALA, was shown to induce apoptosis in MDA-MB-231 cells more effectively than AGAP alone, in addition to inhibiting cell invasion, an effect not observed with AGAP by itself. The authors suggested that fusion of biotoxins with tumor target domain could provide a simple yet effective way to delivery of peptide or protein drugs [17].

Six anticancer peptides have been isolated from snake venoms. Among them, two peptides derived from *Naja naja* demonstrated cytotoxic activity against breast cancer cell lines. Cytotoxin-II showed activity against MCF-7 cells, with an IC_50_ value of 4.18 μg/mL [18], whereas NN-32 exhibited cytotoxic effects against both MCF-7 and MDA-MB-231 cells, with IC_50_ values of 2.5 μg/mL and 6.7 μg/mL, respectively [19]. In addition, NN-32 displayed lower cytotoxicity toward non-tumoral MCF-10A breast epithelial cells (IC_50_ = 25 μg/mL), suggesting a degree of selectivity toward tumor cells [19]. Ebrahim and coworkers demonstrated that Cytotoxin-II primarily induces apoptotic cell death in MCF-7 cells. Microscopy analyses revealed morphological alterations consistent with apoptosis, including chromatin condensation, increased Annexin V staining, caspase-9 activation, and accumulation of cells in the sub-G1 phase, although minor necrotic features were also observed. Furthermore, Cytotoxin-II exhibited greater cytotoxic potency than cisplatin, which presented an IC_50_ value of approximately 28.02 μg/mL in the same cell line [18]. Although the mechanism of cell death induced by NN-32 has not yet been elucidated, the peptide may share a mechanism similar to Cytotoxin-II, since both peptides possess identical N-terminal amino acid sequences. Therefore, they were suggested to belong to the same toxin family [18,19].

Another snake-derived peptide, EVP50, was developed from Lachesicidin, a peptide isolated from *Lachesis muta* venom. This short peptide, containing fewer than 15 amino acid residues, was conjugated to rhodamine B (RhoB-EVP50) and demonstrated activity against both the MDA-MB-231 and MCF-7 cell lines, with IC_50_ values of 30 μM and 20 μM, respectively. In addition, RhoB-EVP50 promoted extracellular calcium influx and intracellular calcium release in MCF-7 cells [20].

The Rusvikunin complex, isolated from *Daboia russelii russelii* venom, is composed of two associated Kunitz-type serine protease inhibitor peptides, Rusvikunin and Rusvikunin-II. This complex demonstrated cytotoxic effects against the MCF-7 breast cancer cell line at concentrations of 10 μg/mL; however, its mechanism of action remains unclear [21]. Vicrostatin (VCN), developed from contortrostatin, a disintegrin isolated from the venom of the viper *Agkistrodon contortrix contortrix* [22], is a long peptide with desintegrin activity that showed activity against MDA-MB-435 cell line with an IC_50_ 0.48 μM. An in vivo study showed that VCN binds to tumor in the xenograft model, using this cell line, in a model with radioactive ^64^Cu linked to peptide as a probe to evaluate this binding [23]. Moreover, some studies were performed using a combination (not specifying which peptides, proportions, and concentrations) between peptides from snake and scorpion. ICD-85 is a formulation created from peptides of *Agkistrodon halys* and *Hemiscorpius lepturus* venoms that demonstrated anticancer activities against breast cell lines such as MCF-7 and MDA-MB-231 [24,25]. Furthermore, an evaluation of toxicity against the non-tumor fibroblast cell line showed that ICD-85 formulation was not toxic at twice the toxic concentration for MCF-7 cells (36.45 μg/mL). The action mechanism appears to be by apoptosis, with evidence to caspases activation, but in high concentrations, perhaps necrosis can occur, based on evidence of lactate dehydrogenase release [25].

Bees are also an important source of peptides with activity against breast cancer cells. Among the seven studies identified, four investigated melittin, the best-characterized peptide from the genus *Apis*. One study demonstrated that crude venom from *Apis mellifera* exhibited cytotoxic activity against MDA-MB-231 breast cancer cells, with IC_50_ values of 6.25 μg/mL and 3.125 μg/mL after 24 and 48 h of treatment, respectively. In addition, the venom induced cell shrinkage, morphological irregularities, cell detachment, and severe membrane damage, ultimately leading to apoptosis [26]. Two studies investigated the activity of isolated melittin against breast cancer cell lines. In one study, melittin exhibited potent cytotoxic activity against MCF-7, MDA-MB-231, and MDA-MB-435 cells, with IC_50_ values below 5 μg/mL. However, a similar IC_50_ value was observed in non-tumoral MCF-10A cells (2.94 μg/mL), indicating limited selectivity toward cancer cells [27]. Another study reported an IC_50_ value of 16.67 μg/mL against MCF-7 cells using melittin isolated from *Apis cerana* [28]. Mechanistically, melittin induces cell death through both apoptotic and necrotic pathways and reduces AKT phosphorylation [27].

Given the extensive investigation of melittin as an anticancer peptide, several studies have explored its incorporation into nanoparticle-based delivery systems to improve its therapeutic potential. One study evaluated melittin-loaded carbon nanoparticles in vitro and found no significant improvement in anticancer activity compared with free melittin. However, the lack of toxicity evaluation in non-tumoral cells prevented assessment of whether nanoparticle encapsulation could improve the selectivity profile of the peptide. Mechanistic studies performed in MDA-MB-231 and MCF-7 cells suggested that melittin-loaded carbon nanoparticles induced predominantly necrotic cell death at concentrations of 10 μg/mL, although caspase activation was also observed, indicating a possible contribution of apoptotic pathways [29]. A different nanoparticle formulation based on a perfluorooctyl bromide (PFOB) emulsion was evaluated in both breast cancer and melanoma models. In melanoma-bearing mice implanted with C32 cells, melittin-loaded PFOB nanoparticles induced apoptosis and significantly inhibited tumor growth. Similarly, treatment with melittin-loaded nanoparticles (2.5 mg/kg, i.v., every third day for five doses) markedly suppressed tumor growth in a xenograft model of MDA-MB-435 breast cancer [30]. 

Other studies involving bee peptides were performed with two peptides from *Apis mellifera*, the Promelittin that went from immature melittin sequence and Apamin. The first one, Promelittin, was active against MCF-7 cells in low concentrations (IC_50_ 6.1 μM) and was active against non-tumor cells like erythrocytes only in higher concentrations (IC_50_ 72.2 μM) [31]. The other one, Apamin (APA), was used in APA-functionalized emulsomes to potentiate the cytotoxicity of Ellagic Acid (a polyphenol from ellagitannin family) against MCF-7. It was observed that the use of the peptide APA in the formulation improved the cytotoxicity of Ellagic Acid to MCF-7 (IC_50_ 5.47 μg/mL), the cell penetration and accumulation [32]. Also was described that Apamin combined to Ellagic Acid acts by apoptosis, according to flow cytometry evidence. In this analysis, the subdiploid DNA content of cells indi-cate late apoptosis, in addition, some apoptotic markers were found in high levels [29,32].

Another important source of peptides with anti-breast cancer activity are spider toxins, particularly those isolated from *Lycosa erythrognatha*, *Lycosa singorensis*, *Chilobrachys jingzhao*, and *Latrodectus tredecimguttatus*. The *L. erythrognatha* synthetic peptides derivated from LyeTx I peptide were investigated against some cancer cells lines. LyeTx I-b is a peptide derived from LyeTx I, which lacks an aminoacid residue of histidine, was tested against triple-negative breast cancer cells including human MDA-MB-231 (IC_50_ values ranged from 5.7 to 2.4 μM) and mouse model of triple-negative-4T1 cells (IC_50_ 6.5 μM) [33,34]. Cytotoxicity also was observed to MCF-7 (IC_50_ 7.3 μM) [33]. Moreover, LyeTx I-b exhibited a synergistic effect when combined with cisplatin on MDA-MB-231 cells. This combination induced autophagy through regulation of the AKT/ERK signaling pathway, suggesting a potential mechanism underlying the enhanced antitumor activity. In addition, cytotoxicity assays performed in non-tumoral HEK-293 cells revealed an IC_50_ value of 7.8 μM [34]. Moreover, an in vivo study was performed using the isogenic breast cancer 4T1 model. It was found an activity with 5 mg/kg (subcutaneous or intratumor injection), where tumors decreased their weight, volume, and inflammatory response. Interesting was the reduction of the number of metastatic nodules in the lungs, demonstrating the antimetastatic potential of this peptide [33]. Lycosin-I, isolated from *Lycosa singorensis*, was evaluated for its cytotoxic activity, exhibiting IC_50_ values below 40 μM against MCF-7 cells and below 20 μM against MDA-MB-231 cells. However, no additional evidence was provided regarding the underlying mechanism of cell death induced by the peptide in these cell lines [35]. Another peptide, Latroeggtoxin-V, purified from the spider *Latrodectus tredecimguttatus* eggs, showed activity against MDA-MB-231 cell line (IC_50_ 43 μM) and toxicity against HEK-293 cells with an IC_50_ higher than 80 μM. Mechanism studies in MDA-MB-231 shows that Latroeggtoxin-V reduced cell proliferation and migration, inhibited Na^+^/K^+^-ATPase and acted by apoptosis, with evidence of cell cycle arrest in G0/G1 cell cycle, as well as cells exhibiting karyopyknosis upon morphological analyses [36].

Another spider-derived peptide, JZTX-14, isolated from *Chilobrachys jingzhao*, exhibited activity against MDA-MB-231 breast cancer cells. Electrophysiological studies demonstrated that JZTX-14 inhibits Nav1.5-mediated sodium currents in a concentration-dependent manner, with an IC_50_ value of 401 nM for sodium channel inhibition. Although the peptide did not affect cell proliferation, it significantly inhibited cell migration and invasion, exhibiting an IC_50_ value of 6 μM in invasion assays. RNA-seq analysis further revealed substantial transcriptional modulation following JZTX-14 treatment, with 184 genes upregulated and 154 downregulated. Among the most affected genes were ANGPTL4, GJB3, FBXO2, and HMGN5, which were downregulated, whereas IL-24 and SERPINB2 were upregulated; these findings were subsequently validated by quantitative PCR (qPCR) [37].

Other animals such as wasps and snails are sources of anti-breast-cancer peptides. Mastoparan from wasp had activity against MDA-MB-231 cells with IC_50_ around 25 μM and in vivo experiments showed activity comparable to antitumor agent Gemcitabine as well an indicative to synergism when both were combined. There is evidence that Mastoparan kills MDA-MB-231 cells by lysis, as observed in experiments of lactate dehydrogenase leakage and microscopy [38]. A Conotoxin from marine cone snail *Conus loroisii* showed activity against MCF-7 cells (IC_50_ 32 μg/mL), although there is no information about its activity on non-tumoral cells. In vivo tests were performed to evaluate toxicity of this conotoxin using the Brine Shrimp Lethality Assay (BSLA), with an effective dose for 50% of the population (DE_50_) of 30 μg/mL. Microscopy data suggest apoptosis as cell death mechanism due the chromatin condensation after treatment with Conotoxin [39] even though more experiments should be performed to verify this indication. Table 1 summarizes some data of peptides acting in breast cancer.

**Table 1:** Anti-breast-cancer activity of toxin-derived peptides and possible mechanisms of action.

Animal and Specie	Peptide	In Vitro Activity (IC_50_)	In Vivo Treatment	Effects and Mechanism of Action (When Investigated)	SI	References
Scorpions						
*Androctonus australis*	KAaH1	>50 μg/mL (inhibitis cell migration in MDA-MB-231)	-	Blocks ion channels (Kv1.1 and Kv1.3).	-	[12]
*Androctonus australis*	KAaH2	>50 μg/mL (inhibitis cell migration in MDA-MB-231)	-	Blocks ion channels (Kv1.1 and Kv1.3).	-	[12]
*Tityus serrulatus*	ε-Ahx-TsAP-1	20 μM (MDA-MB-231) and 10 μM (MCF7)	-	Induces apoptosis.	-	[13]
*Leiurus quinquestriatus*	Chlorotoxin	5 μM (MDA-MB-231, MCF7 and T47D)	- (i.v.)	In vitro: Inhibits tumor proliferation, migration, and invasion. Binds to ERα. Decreases the expression levels of Erα (MCF-7) and VASP (MCF-7 and MDA-MB-231). In vivo: inhibits MCF-7 tumor growth and weight.	-	[14]
*Mesobuthus martensii Karsch* (*Buthus martensii Karsch*)	BmKn-2	30 µg/mL (in canine brest metastatic cells CHMp-5b) and 54 µg/mL (in canine brest non metastatic cells CHMp-13a	-	Induces apoptosis (Bax overexpression and Bcl-2 suppression).	-	[15]
*Buthus martensii Karsch* (*Buthus martensii Karsch*)	BmK AGAP	50 μM (MDA-MB-231) and 40 μM (MCF7)	0.5 or 1.0 mg/kg (i.p.)	In vitro: inhibits cell proliferation and EMT. Inhibits the NF-κB and Wnt/β-catenin signaling pathways Suppress Nav 1.5 channel. In vivo: inhibits breast tumor growth, stem-like features and EMT.	-	[16]
*Buthus martensii Karsch* (*Buthus martensii Karsch*)	AGAP linked by N-terminal to ATF peptide (ALA protein)	5–10 μg/mL (MDA-MB-231), >100 μg/mL (MCF7) and >100 μg/mL (HEK-293)	-	Inhibits tumor cell invasion. Induces apoptosis.	-	[17]
Snakes						
Mix between peptides from snake *Agkistrodon halys* scorpion *Hemiscorpius lepturus*	ICD-85	36.45 µg/mL (MCF7), active without values dermined agaisnt MDA-MB-231 and >80 µg/mL (HDF)	-	Induces apoptosis and possibility necrosis.	-	[25]
*Naja naja*	NN-32	6.7 μg/mL (MDA-MB-231), 2.5 μg/mL (MCF7) and 25 μg/mL (MCF10A)	-	Induces apoptosis and possibility necrosis.	-	[19]
*Naja naja oxiana*	Cytotoxin-II	4.18 μg/mL (MCF7) and 18.12 μg/mL (MCF10A)	-	Induces apoptosis.	-	[18]
Vipers						
*Lachesis muta*	EVP50	30 μM (MDA-MB-231) and 20 μM (MCF7)	-	Induces calcium influx and intracellular calcium release.	-	[20]
*Daboia russelii russelii*	Rusvikunin complex	- (MCF7)	-	Induces cancer cell cytotoxicity.	-	[21]
*Agkistrodon contortrix contortrix*	VCN	0.48 μM (MDA-MB-435)	100–150 μCi of radioactive marked peptide to evalaute its tumor binding capacity, no cytotoxity evaluation	In vitro: Displays desintegrin activity. In vivo: Binds selectivily to tumor cells.	-	[23]
Bees						
*Apis mellifera*	Crude venom	6.25 μg/mL and 3.125 μg/mL after 24 and 48 h, respectively (MDA-MB-231)	-	Induces morphological tumor cell alterations, including cell shrinkage, cellular shape irregularity, cellular detachment and membrane demage.	-	[26].
*Apis mellifera*	Melittin	3.24 μg/mL (MDA-MB-231) 4.03 μg/mL (MDA-MB-435) 4.68 μg/mL (MCF7) and 2.94 μg/mL (MCF10A)	-	Induces apoptosis.	-	[27]
*Apis cerana*	Melittin	16.67 μg/mL (MCF7)	-	Induces apoptosis.	-	[28]
*Apis mellifera*	Melittin in carbon nanoparticles	<10 μg/mL (MDA-MB_231 and MCF7)	-	Induces necrosis.	-	[29]
*Apis mellifera*	Melittin in nanoparticles of perfluorooctyl bromide (PFOB)	-	1 mg/kg (i.v.)	Inhibits tumor growth.	-	[30]
*Apis mellifera*	Promelittin	6.1 μM (MCF7) and 72 μM (erythrocytes)	-	-	6.7	[31]
*Apis mellifera*	Apamin (combined with egalic acid)—APA	5.47 μg/mL (MCF7)	-	Induces apoptosis.	-	[32]
Spiders						
*Lycosa erythrognatha*	LyeTxI-b	2.47 μM (MDA-MB-231), 7.34 μM (MCF7), 6.5 μM (4T1) and 7.88 μM (HEK-293)	5 mg/kg (systemic or s.c.injections)	Induces autophagic cell death in assocition synergistic with Cisplatin/regulation of AKT/ERK pathway.	3.19 (HEK/MDA)	[33,34]
*Lycosa singorensis*	Lycosin-I	MCF-7 (<40 μM) and MDA-MB-231 (<20 μM)	-	-	-	[35]
*Latrodectus tredecimguttatus*	Latroeggtoxin-V	43 μM (MDA-MD-231) and >80 μM (HEK-293)	-	Inhition of cell proliferation, migration, Na^+^/K^+^-ATPase and apoptosis induction, cell cycle arrest (G0/G1) and karyopyknosis.	-	[36]
*Chilobrachys jingzhao*	JZTX-14	6 μM (MDA-MB-231, invasion assay)	-	Inhibits Nav1.5-mediated sodium currents (IC_50_ = 401 nM), suppresses migration and invasion, and modulates the expression of genes associated with tumor progression.	-	[37]
Other species						
Wasp	Mastoparan	<25 μM (MDA-MB-231)	6 mg/kg (i.p.)	Induces cell lysis.	2–6 fold	[38]
Marine cone snail *Conus loroisii*	Conotoxin	32 μg/mL (MCF7)	Brine Shrimp Lethality Assay (BSLA)	Induces apoptosis.	-	[39]

AGAP: analgesic–antitumor peptide; Erα: estrogen receptor alpha; i.p.: intraperitoneal; i.v.: intravenous; s.c.: subcutaneous; SI: selectivity index; VASP: vasodilator stimulated phosphoprotein; VCN: vicrostatin.

### 1.2. Cervical Cancer

Active toxic peptides from animals, as those derived from bees, snakes, toads, spiders, scorpions, mollusks, and fish, have also become promising in the treatment of cervical cancer (CC) [40,41] (Table 2).

In the case of bees, both the crude venom and several derived peptides show antitumor activity in CC [26,41,42,43,44,45,46,47]. Despite being composed of several bioactive molecules, encompassing melittin, adolapin, apamin, mast cell degranulating (MCD) peptide, phospholipase A2 (PLA2), hyaluronidase, and bioactive amines, the majority of bee’s crude venom is composed of melittin (40–60%), a peptide of 26 amino acid residues [48,49].

The antitumor effects of melittin in cervical cancer were initially investigated in CaSki cells. The peptide inhibited cell proliferation in a concentration-dependent manner, reducing proliferation by approximately 35% and 50% at concentrations of 3 and 4 μg/mL, respectively. In addition, melittin was evaluated for its ability to inhibit cell migration induced by epidermal growth factor (EGF) and angiogenesis, through the suppression of vascular endothelial growth factor (VEGF) and hypoxia-inducible factor 1-alpha (HIF-1α) expression. Furthermore, evidence indicates a reduction in protein synthesis due to the inhibition of the ERK, mTOR, and p70S6K signaling pathways [44]. Subsequently, similar results were reported for melittin isolated from *Apis mellifera meda*, with analysis performed on other CC cell lines (HeLa and C33A). The most pronounced antitumor activity was observed in HeLa cells, where an IC_50_ of 1.7 μg/mL was recorded after 24 h, along with flow cytometry data suggesting apoptosis as the primary mechanism of cell death [45]. In the case of melittin derived from *Apis mellifera syriaca*, an IC_50_ of 19.7 μg/mL was observed against HeLa cells, although no mechanistic analysis has yet been conducted for this type of melittin [46].

The antitumor activity of melittin in HeLa cells has also been evaluated following chemical modifications, such as melittin-DMMA (2,3-dimethyl maleimide), [42]. This modification reduced the peptide’s hemolytic activity against erythrocytes while maintaining its cytotoxic effects on HeLa cells at concentrations above 20 μg/mL. In in vivo biocompatibility experiments using zebrafish larvae, no serious malformations or embryo deaths were observed in neutral or alkaline media. However, no evidence regarding the mechanism of cell death was provided [42]. Another modification involved the association of melittin with a pegylated graphene oxide nanocomposite carrier (PEG-GO-Fe_3_O_4_/melittin). This system protected melittin from degradation and denaturation, promoting a slow and sustained release of the peptide, which resulted in enhanced antitumor activity against HeLa cells. The effective concentrations for PEG-GO-Fe_3_O_4_/melittin were 13 μg/mL/5 μg/mL [43].

Crude venom from *Apis mellifera* was tested against HeLa cells, demonstrating cytotoxic activity at a concentration of 6.25 μg/mL, which resulted in 46% cell viability. These findings suggest an IC_50_ below 6.25 μg/mL, with morphological evidence indicating apoptosis as the likely mechanism of cell death [26].

Except for melittin, few peptides of animal origin have had their antitumor activity evaluated in this cancer model [50,51,52,53]. One of the first peptides tested for CC was triflavin, purified from the venom of the snake *Trimeresurus flavoviridis*. Triflavin is a trigramin-like Arg-Gly-Asp (RGD) peptide composed of 70 amino acid residues in its primary structure, including 12 cysteines. It is a potent inhibitor of platelet aggregation, and, in in vitro analyses, reduced platelet aggregation induced by HeLa cells (1 μg/mL) in platelet-rich heparinized plasma in a concentration-dependent manner. Based on this evidence, the authors concluded that triflavin can prevent metastasis formation by inhibiting platelet aggregation induced by HeLa cells [53]. Crude venom from *Bothrops jararaca* and *Bothrops erythromelas* demonstrated significant anticancer activity in cervical carcinoma cell lines. In vitro exposure of SiHa and HeLa cells lines to crude venom (12.5–50 μg/mL, 24–48 h) led to dose-dependent reductions in cell viability, Flow cytometry with annexin V/PI showed that up to 90–95% of tumor cells underwent apoptosis after 48 h treatment with 50 μg/mL venom, comparable to cisplatin control. 4′,6-diamidino-2-phenylindole (DAPI) staining confirmed chromatin condensation, nuclear fragmentation, and apoptotic bodies. Additionally, cell cycle analysis demonstrated arrest in G0/G1 phase, preventing progression to S and G2/M [54].

Lycosin-I peptide, purified from the venom of the spider *Lycosa singorensis*, has the ability to penetrate cell membranes. To enhance its antitumor activity, it was conjugated to gold nanoparticles (LGNP) and gold nanorods (LGNR). The results demonstrated that the lycosin-I conjugate, LGNP, significantly increased the cell penetration capacity of the nanoparticles against HeLa cells. Moreover, LGNP exhibited inhibitory activity against HeLa cells with more than 50% inhibition at a concentration of 12.5 μM, whereas this level of activity was observed against HEK293 cells only at 25 μM. In comparison, lycosin-I alone had an IC_50_ of 10 μM. Evidence of intracellular translocation was associated with both nanoparticle concentration and incubation time. In vivo biodistribution studies using Balb/c mice demonstrated that the accumulation of LGNPs in tumor tissue was approximately eight-fold higher than that of PEG-modified gold nanoparticles, the control nanoparticle formulation, supporting the ability of Lycosin-I to promote selective tumor targeting and accumulation in vivo. Additionally, LGNRs selectively targeted tumor cells and, upon near-infrared irradiation, promoted a photothermal effect that reduced both cell viability in vitro and tumor volume in vivo [51]. However, no mechanistic experiments were conducted in this study.

In line with cell affinity assays to identify cancer cell-binding peptides (CBPs), the peptide buforin IIb, isolated from the poison of the Asian toad *Bufo bufo gargarizans*, demonstrated high affinity for HeLa tumor cells due to its specific binding to gangliosides present on the cancer cell surface. This affinity resulted in significant tumor suppression in vivo experiments. Buforin IIb exhibited high activity against HeLa cells (IC_50_ = 12 μg/mL), while its IC_50_ against normal fibroblasts was 350 μg/mL. Additionally, buforin IIb showed in vivo activity in a xenograft mouse model at a dose of 5 mg/kg. Furthermore, induction of apoptosis through mitochondrial caspase 9 activation was observed, alongside membrane permeabilization mediated by endoplasmic reticulum (ER) stress, with cytochrome C release [55]. Moreover, the peptide and NFTGDSIPC(+57.02)R, one of the 76 CBPs isolated from the poison of the same amphibian, demonstrated antitumor activity by inhibiting HeLa cell growth (IC_50_ = 84.51 μg/mL). They also exhibited the capacity for internalization and accumulation in the nucleus of HeLa cells, while the poison peptide mixture was visualized in the cytoplasm, nucleus, and other organelles [52].

Scorpion venom presents only one peptide with documented activity against CC. The peptide HL-10, isolated from *Hemiscorpius lepturus*, demonstrated cytotoxicity against HeLa cells with an IC_50_ of 40.0 μM. The suggested mechanism of cell death is apoptosis, supported by evidence from flow cytometry, upregulation of Bax, p53, caspase-3, and PTEN gene expression. Additionally, an increase in reactive oxygen species (ROS) levels was also observed [56].

The peptide mix ICD-85 derived from *Agkistrodon halys* snake and *Hemiscorpius lepturus* scorpion, exhibits cytotoxic and pro-apoptotic activity against human cervical carcinoma HeLa cells. When encapsulated into alginate-based nanoparticles (ICD-85 NPs, ~200 nm), the formulation enhanced antiproliferative efficacy. In MTT assays (72 h), the IC_50_ decreased from 25 ± 2.9 μg/mL for free ICD-85 to 15.5 ± 2.4 μg/mL for ICD-85 NPs, indicating greater potency. Morphological analysis confirmed membrane destabilization and apoptotic features after exposure to 28 μg/mL of either free or nanoparticle-encapsulated ICD-85. Caspase-8 colorimetric assay demonstrated significant activation following treatment, with ICD-85 NPs inducing stronger apoptosis than free ICD-85, consistent with an extrinsic apoptotic pathway. Interestingly, lactate dehydrogenase (LDH) assays showed that free ICD-85 produced higher necrotic damage, while ICD-85 NPs reduced necrosis but favored apoptotic induction, likely due to sustained release [57].

The venom of the ray *Dasyatis sephen*, extracted from tail spines, exhibits antiproliferative activity against HeLa cells. Crude venom reduced cell viability in a dose-dependent manner, achieving up to 80–81% inhibition at 16–20 μg/mL. Mechanistic investigations revealed that venom treatment markedly increased intracellular ROS levels (12–75% elevation across 4–16 μg/mL), enhanced lipid peroxidation markers (TBARS, conjugated dienes, lipid hydroperoxides), and disrupted the mitochondrial membrane potential (depolarization reduced to 10% of control at 16 μg/mL). Dual AO/EtBr staining confirmed morphological hallmarks of apoptosis, with apoptotic cell rates rising to 74–78% at 12–16 μg/mL [58].

**Table 2:** Anti-cervical-cancer activity of toxin-derived peptides and possible mechanisms of action.

Animal and Specie	Peptide	In Vitro Activity (IC_50_)	In Vivo Treatment	Effects and Mechanism of Action (When Investigated)	SI	References
Bees						
Bee *Apis mellifera*	Melittin	>3.0 μg/mL (CaSki)	2 μg/mL) (matrigel plug assay)	Reduces angiogenesis and tumor progression via inhibition of EGF-induced VEGF secretion.	-	[44]
Bee *Apis mellifera*	Melittin	1.7 μg/mL (HeLa)	-	Induces apoptosis.	-	[44,45]
Bee *Apis mellifera syriaca*	Melittin	19.7 μg/mL (HeLa)	-	Probably induces apoptosis.	-	[46]
Bee	Melittin-DMMA	>20 μg/mL (HeLa)	--	Induces apoptosis.	-	[42]
Bee *Apis mellifera*	PEG-GO-Fe_3_O_4_ Melittin	PEG-GO-Fe_3_O_4_/MEL (13 μg mL^−1^/5 μg mL^−1^) (HeLa)	-	Inhibits cell proliferation. Induces pores membrane formation.	-	[43]
Bee *Apis mellifera*	Crude venom	<6.25 μg/mL (HeLa)	-	Induces apoptosis.	-	[26]
Snakes						
Snake *Trimeresurus flavoviridis*	Triflavin (RGD—Arg-Gly-Asp) peptide	1 μg/mL (HeLa)	-	Inhibits platelet aggregation.	-	[53]
Snake *Bothrops jararaca and Bothropserythromela*	Crude venom	12.5 μg/mL (HeLa and SiHa)	-	Inhibits cell proliferation. Induces apoptosis.	-	[54]
Mix between peptides from snake *Agkistrodon halys* and scorpion *Hemiscorpius lepturus*	ICD-85 NP (Nanoparticles)	15.5 μg/mL (HeLa)	-	Induces higher levels of caspase 8. Induces apoptosis.	-	[57]
Toads						
Toad *Bufo bufo gargarizans*	Buforin IIb	12 μg/mL (HeLa) and 350 μg/mL (Fibroblasts)	5 mg/kg (i.v.)	Induces apoptosis induction by a mitochondria-dependent pathway. Promotes tumor suppression.	-	[55]
Toad *Bufo bufo gargarizans*	Buforin IIb and NFTGDSIPC(+57.02)R	84.51 μg/mL (HeLa)	-	Induces cancer cell cytotoxicity.	-	[52]
Other species						
Spider *Lycosa singorensis*	LGNP	Aprox. 12.5 μg/mL (HeLa)	10 mg/kg (i.v.)	LGNP was significantly more effective in internalizing tumor cells. Infrared light irradiation in cells that internalized lysine-LGNP resulted in cell death both in vitro and in vivo.	-	[51]
Scorpion *Hemiscorpius lepturus*	HL-10	40.0 μM	-	Induces apoptosis and increases ROS.	-	[56]
Ray *Dasyatis sephen*	Crude venom	16–20 μg/mL 16 μg/mL (Inhibits aproximately 80% of HeLa cells)	-	Reduces proliferation via oxidative mechanism. Promotes alteration in the mitochondrial membrane Induces apoptosis.		[58]

i.v.: intravenous; LGNP: lycosin-I-conjugated spherical gold nanoparticles; SI: selectivity index.

### 1.3. Lung Cancer

Lung cancer is the most prevalent cause of cancer-related mortality worldwide, representing the leading cause of death among both men and women and posing a significant public health challenge [59]. Capable of acting on different cellular mechanisms, different animal toxin peptides exhibit anticancer activity and may be potential candidates for lung cancer treatment. Table 3 provides a comprehensive summary of the principal findings regarding the anticancer activity of toxin-derived peptides and crude venoms and poisons encompassing key in vitro and in vivo studies, and the associated cellular mechanisms.

LVTX8, an amphipathic alpha-helical cell-penetrating peptide purified from *Lycosa vittata* spider venom, showed promising anticancer effects in both in vitro and in vivo studies. By inducing apoptosis-mediated tumor cell death, LVTX8 effectively suppressed lung cancer cell proliferation, migration, and invasion. With an IC_50_ of 8 μM in A549 and H460 lung cancer cells, and 19 μM in non-cancer cells, this peptide exhibited a higher selectivity for tumor cells. In vivo, treatment with 10 mg/kg of LVTX-8 prevented metastasis of A549 and H460 cells and significantly inhibited tumor growth in nude mice [60]. Similarly, Dermaseptin-PP, a novel peptide from *Phyllomedusa palliata* frog skin, exhibits low toxicity in non-tumoral cells. It demonstrates selective cytotoxicity against H157 cancer cells, with an IC_50_ around 1.55 µM, inducing significant membrane disruption at concentrations of 10^−4^ M, while exerting minimal effects on non-tumoral HMEC-1 cells. In vivo assays further confirm its anticancer potential, showing that Dermaseptin-PP inhibits tumor growth, especially in higher dose, and promotes cancer cell death by apoptosis [61]. Additionally, other peptides derived from skin secretions of the frogs within the *Phyllomedusa genus*, namely phylloseptin-Pta and phylloseptin-Pha, demonstrated greater potency against H157 lung cancer cells than against non-tumoral HMEC-1 cells. The peptides exhibited IC_50_ values of 6.73 μM and 14.10 μM, respectively, in H157 cells, whereas HMEC-1 viability remained largely unaffected at concentrations up to 10 μM. Phylloseptin-PTa showed marked cytotoxicity only at the highest concentration tested (100 μM), while phylloseptin-PHa produced only a modest reduction in HMEC-1 viability at this concentration [62].

Beyond amphibian-derived peptides, venom components from reptiles have also exhibited promising lung anticancer effects. Crotoxin, a neurotoxin derived from the venom of the South American rattlesnake *Crotalus durissus terrificus*, has demonstrated cytotoxic activity against the lung squamous carcinoma cell line SK-MES-1, with an IC_50_ of 25.13 μg/mL. This effect is mediated through the induction of apoptosis and autophagy via activation of the p38 MAPK signaling pathway [63].

Marine-derived peptides further expand the spectrum of venom-based lung anticancer agents. A synthetic peptide isolated from marine snails *Conus californicus* venom, namely s-cal14.1a, acts as a competitive antagonist of acetylcholine nicotinic receptors (nAChRs) expressed in lung cancer cells, leading to activation of effector caspases-3 and -7, and, consequently, cell death by apoptosis [64]. Another study also demonstrated that the synthetic conotoxin Cal14.1a increases Bax expression and decreases H1299 lung cancer cell viability [65]. Given that nAChR signaling is closely associated with lung cancer development and survival [66], targeting this pathway represents a promising therapeutic strategy for lung cancer treatment.

In addition to purified peptides derived from venoms, crude venoms themselves have been extensively studied for their potential therapeutic applications. Despite their high toxicity and rapid action, these complex mixtures have demonstrated promising effects in the treatment of various pathological conditions, including lung cancer. The crude venom of *Conus virgo* snail exhibited antitumor activity against A549 cells (IC_50_ of 74.69), inducing cancer cell morphological changes and DNA fragmentation [67]. Similarly, the crude venom of the sea anemone *Calliactis tricolor* showed cytotoxicity against A549 cells, with an IC_50_ of 60 μg/mL [68]. Peroxiredoxin 6 (PRDX6), a bifunctional protein member of the peroxiredoxin (PRDX) family, has been associated with lung cancer cell growth and development [69]. In this context, a previous study demonstrated that snake venom toxin (SVT) from *Vipera lebetina turanica*, with an IC_50_ of 6.8 μg/mL against A549 and NCI-H460 cells, blocked PRDX6 transcription, possibly through the inactivation of AP-1, a protein complex related with a variety of pro-tumor functions. In in vivo assays, 0.5 or 1 mg/kg of STV significantly increased the expression of cleaved caspase-3 and inhibited tumor growth [70]. The treatment with crude spider and scorpion venom and its fractions derived from *Phlogiellus bundokalbo* and *Androctonus australis Hector*, respectively, also induced lung cancer cell cytotoxicity and apoptotic morphological changes [71,72]. Furthermore, the crude poison derived from the toad *Rhinella horribilis*, with an IC_50_ of 0.031 µg/mL in A549 cells, effectively inhibited cancer cell proliferation and migration while promoted tumor cell death [73].

Toxin-derived compounds have demonstrated significant potential in lung cancer treatment by inhibiting tumor growth, suppressing metastasis, and inducing cancer cell death through apoptosis and autophagy. Key mechanisms include the disruption of membrane integrity, modulation of PRDX6 expression, inactivation of AP-1, and inhibition of nAChR signaling, all of which are critical for lung cancer progression and survival. Despite these promising findings, further research is essential to refine their selectivity, minimize systemic toxicity, and translate these bioactive molecules into clinically viable therapies.

**Table 3:** Anti-lung-cancer activity of toxin-derived peptides and possible mechanisms of action.

Animal and Specie	Peptide	In Vitro Activity (IC_50_)	In Vivo Treatment	Effects and Mechanism of Action (When Investigated)	SI	References
Spiders						
Spider *Lycosa vittata*	LVTX8	Approximately 8 μM (A549 and H460)	10 mg/kg (i.p.)	In vitro: Induces apoptosis. In vivo: Prevents metastase and reduces tumor growth.	-	[60]
Spider *Phlogiellus bundokalbo*	Crude venom	25.12 μg/mL (A549)	-	Activates caspase 3 and 7. Induces morphological tumor cell alterations including membrane blebbing, cell shrinkage and nuclear condensation.	-	[71]
Toads						
Frog *Phyllomedusa palliata*	Dermaseptin-PP	1.55 μM (H157)	20 μL from 8, 6, 4 mM (i.t.)	In vitro: Induces apoptosis. In vivo: Exhibits dose-dependent anti-tumor activity without significant hepatopulmonary side effects.	-	[61]
Frog *Phyllomedusa tarsius*	Phylloseptin PTa	6.73 μM (H157)	-	Induces cancer cell cytotoxicity.	-	[62]
Frog *Phyllomedusa hypochondrialis*	Phylloseptin Pha	14.10 μM (H157)	-	Induces cancer cell cytotoxicity.	-	[62]
Toad *Rhinella horribilis*	Crude secretion	0.031 µg/mL (A549)	-	Increases ROS release. Induces apoptosis.	-	[73]
Snail						
Snail *Conus californicus*	s-call4.1a	-	-	Activates caspase 3 and 7 Decreases expression of pro-survival protein NFκB-1.	-	[64]
Snail *Californiconus caifornicus*	Cal14.1b	-	-	Increases Bax expression. Induces cell cytotoxicity.	-	[65].
Snail Conus virgo	Crude venom	74.69 μg/mL (A549)	-	Induces DNA fragmentation and morphological tumor cell alterations, including cell shrinkage and cellular blabbing.	-	[74]
Other species						
Snake *Crotalus durissus terrificus*	Crotoxin	25.13 μg/mL (SK-MES-1)	-	Induces autophagy and cell death by apoptosis.	-	[63]
Anemone *Calliactis* *tricolor*	Crude venom	60 μg/mL (A549)	-	Induces cell cytotoxicity.	-	[68]
Viper *Lebetina turanica*	Crude venom	6.8 μg/mL (A549 and NCI-H460)	0.5 mg/kg and 1 mg/kg (i.p.)	In vitro: Increases Bax, cleaved form of caspase-3, -8, -9, and p21 and p53 expressions. Induces apoptosis. In vivo: Inhibited tumor volume and growth.	-	[70]
Scorpion *Androctonus australis hector*	Crude venom and F3 fraction	396.60 μg/mL (crude venom) and 27.05 μg/mL (F3 fraction) (NCI-H358)	-	Activates caspase 3. Induces morphological tumor cell alterations including DNA fragmentation, nucleus shrinkage, chromatin condensation and formation of apoptotic bodies. Produces ROS and nitrite. Decreases SOD and catalase activity and GSH levels.	-	[72]

i.p.: intraperitoneal; i.t.: intratumoral; ROS: reactive oxygen species; SI: selectivity index; SOD: superoxide dismutase.

### 1.4. Glioma

Glioma is the most common primary tumor of the central nervous system. The current gold standard treatment consists of a radiotherapy regimen combined with temozolomide, an alkylating agent, known as the Stupp protocol [75]. However, the improvement in survival is often limited due to the development of resistance to temozolomide. In this context, peptides derived from animal toxins have emerged as a promising source of novel antitumor agents against glioma. Venoms from scorpions, spiders, snakes, jellyfish, and bees, both as crude fractions and purified peptides, have been investigated for their effects on glioma and are summarized in Table 4.

Chlorotoxin (CTX), a small neurotoxin isolated from the venom of the scorpion *Leirus quinquestriatus* [76], already cited before. is capable of crossing the blood–brain barrier (BBB). Interestingly, when injected into vertebrates, no apparent signs of toxicity have been observed. Although this peptide does not exhibit cytotoxic effects on D54-MG cells, it has been shown to inhibit the chloride channel CLC-3 on these cells (at 300 nM), thereby preventing the growth and invasion of glioblastoma cells [77].

Some studies have explored the use of CTX-based delivery systems to improve the targeting of chemotherapeutic agents to glioma cells. One example is a liposomal formulation containing doxorubicin conjugated to a human IgG Fc–CTX fusion protein (M-CTX-Fc-L-Dox). In U251MG-P1 glioblastoma cells, this formulation exhibited an IC_50_ value of 0.17 μM, comparable to that observed for free doxorubicin (IC_50_ ≈ 0.20 μM). Although no significant difference in cytotoxicity was detected in vitro, M-CTX-Fc-L-Dox demonstrated enhanced cellular uptake and improved antitumor activity in vivo, highlighting its potential as a targeted drug-delivery system [78]. Additionally, in vivo experiments demonstrated that a 10 mg/kg dose was effective against U251MG-P1 tumor cells in a BALB/c mouse model, with the formulation proving more efficient than free doxorubicin. The mechanism appears to involve interaction with metalloproteinase-2 (MMP-2), which facilitates the internalization of doxorubicin into the cells. In vivo, M-CTX-Fc-Dox exhibited an inhibitory effect on tumor growth, likely due to the combined contribution of passive targeting through the enhanced permeability and retention (EPR) effect and active targeting via receptor-mediated endocytosis [78].

The Lqh-8/6 peptide, a natural analogue of chlorotoxin derived from the venom of *Leiurus quinquestriatus hebraeus*, was chemically synthesized, yielding two variants: one biotinylated and another designed for click chemistry to facilitate conjugation with doxorubicin. The resulting hybrid molecule was evaluated in vitro on two glioma cell lines: rat F98 and human U-87 glioblastoma, demonstrating significant cytotoxic effects in U87 (IC_50_ = 16.6 μM), F98 (IC_50_ = 0.84 μM), and RG-2 (IC_50_ = 4.14 μM) cells. Mechanistic investigations provided robust evidence that the predominant mode of cell death was apoptosis, as indicated by caspase-3 activation, Bax upregulation, and nuclear fragmentation, which were confirmed through microscopy. Moreover, studies using rat brain slices previously inoculated with F98 cells into the striatum showed that the tumor regions corresponded to areas where cancer cells were marked by the Lqh-8/6 analogue [79].

The voltage-dependent potassium channels (Kv1–Kv12) and their respective subtypes have been implicated in cancer progression. KAaH1 and KAaH2 are two non-toxic, purified polypeptides isolated from the scorpion *Androctonus australis Hector*, differing by two amino acids at positions 26 (Ser/Phe) and 29 (Gln/Lys). In glioblastoma cell models (U87), treatment with KAaH1 showed no observable effect, whereas KAaH2 induced a significant inhibition of cell proliferation with an IC_50_ of 7.96 μM. This inhibition was attributed to the blockade of voltage-dependent Kv1.1 and Kv1.3 channels. Additionally, KAaH2 treatment resulted in a reduction of phosphorylated forms of EGFR, ERK, AKT, and JNK proteins, indicating that these signaling pathways are involved in the mechanism of action of KAaH2. KAaH1, on the other hand, inhibited U87 cell adhesion to fibrinogen (42%), fibronectin (20%), and poly-Lysine (17%) coated plates, approximately at its IC_50_ value. Moreover, KAaH1 inhibited the migration of U87 cells [12].

Melittin demonstrates activity against glioblastoma cells, with an IC_50_ of 0.274 μM after 72 h in GAMG cells. However, no in vivo experiments were conducted, nor was the toxicity to normal cells evaluated. The proposed mechanism of cell death is apoptosis, inferred from studies involving other cell types, although no direct experimental evidence was provided in this investigation [80].

The crude venom of the jellyfish *Pelagia noctiluca* was subjected to gel filtration chromatography (Sephadex G75 column), yielding four fractions (F1–F4). Fractions F1 and F2 inhibited the adhesion of U87 glioblastoma cells to fibrinogen, with IC_50_ values of 5 μg/mL and 64 μg/mL, respectively, suggesting that these fractions may interact with adhesion receptors other than integrins. In cell viability assays, the crude venom exhibited an IC_50_ of 180 μg/mL, whereas F1 displayed the highest activity against U87 cells (IC_50_ = 125 μg/mL), indicating greater potency than the unfractionated venom. Fraction F3 also reduced cell viability, with an IC_50_ of 179 μg/mL, while F2 and F4 showed no significant cytotoxic effects. Furthermore, fractions F1 and F3 significantly inhibited U87 cell proliferation after 48 h of incubation, with the antiproliferative effect of F1 being comparable to that of cisplatin (5 μM) [81].

Spider venoms have demonstrated cytotoxic effects on tumors, including gliomas. *Phoneutria nigriventer* and *Lycosa erythrogata* are among the most studied species. Bioactive fractions derived from the crude venom of the spider Phoneutria nigriventer were investigated against human glioblastoma cells (NG97). Following venom fractionation, the SF3 and SF4 subfractions exhibited significant antitumor activity by reducing cell viability and proliferation and inducing apoptosis. These fractions decreased cell viability and proliferation, promoted apoptosis, and potentiated the antiproliferative effects of rapamycin, suggesting their potential as adjuvant therapeutic agents for glioblastoma treatment [82]. Additionally, studies on the toxin Phα1β from *Phoneutria nigriventer* have demonstrated activity at picomolar concentrations against M059J, U-138MG and U251-MG cells (0.3 to 100 pM). The Phα1β peptide and its recombinant form CTK 01512-2 also exhibited in vivo activity in an orthotopic murine glioma model using GL261 cells, with significantly tumor size reduction and promoted neuroimmune activation [83].

The peptide LyeTx I-b, isolated from the venom of *Lycosa erythrognata* spider, exhibits IC_50_ values of 29.20 μM and 20.94 μM against U87 and U373 glioma cells, respectively. On the other hand, it was not cytotoxic against Vero and GM637 cells (IC_50_ > 100 μM). Necroptosis was identified as the principal mechanism of action associated with this peptide, as evidenced by a reduction in cell death following pre-treatment with necrostatin, a necroptosis inhibitor, and confirmed by specific markers using flow cytometry analysis. The anticancer properties of LyeTx I-b appear to be linked to a membranolytic process. Morphological studies using scanning electron microscopy revealed disruption of the plasma membrane in cells treated with LyeTx I-b, including the formation of holes or pores [84]. The crude venoms of the Viperidae species *Cerastes cerastes* (Egypt) and *Cryptelytrops purpureomaculatus* (Thailand) were tested on eight human glioma cell lines, showing potential cytotoxicity against cancerous cells (SHSY5Y and U87MG) as well as a normal cell line (HEK293). After 48 h of treatment, both venoms exhibited activity against the SHSY5Y cell line, with IC_50_ values of 0.12 μg/mL (for *Cerastes cerastes* venom) and 0.25 μg/mL (for *Cryptelytrops purpureomaculatus* venom). Against to U87MG cells, the IC_50_ values were 0.88 μg/mL (for *Cerastes cerastes* venom) and 1.32 μg/mL (for *Cryptelytrops purpureomaculatus* venom). However, challenges remain for their use as therapeutic agents due to toxicity against healthy cells, such as the HEK-293 cell line, making it necessary to determine the exact administration dosage. Further studies are required to elucidate the mechanisms of action of these toxins [85]. Other peptides with activity against U87 cells include CTNanc1 and CTNsen3, isolated from *Naja anchietae* and *Naja senegalensis*, exhibiting IC_50_ values of 36.41 μg/mL and 5.33 μg/mL, respectively. CTNanc1 showed no cytotoxic activity against non-cancerous HUVEC cells, whereas CTNsen3 demonstrated cytotoxicity with an IC_50_ of 3.58 μg/mL. Evidence of the cell death mechanism was provided only for CTNanc1, indicating the induction of apoptosis [86].

Another crude venom tested on the rat glioma cell line (C6) was the venom from *Conus virgo*, a gastropod species, which showed an IC_50_ of 1.5 μg/mL against C6 cells. This venom was found to reduce cell invasion and induce apoptosis through the activation of the p53-dependent apoptosis pathway, as evidenced by the modulation of the Bax and Bcl-2 expression ratio [74].

**Table 4:** Anti-glioma activity of toxin-derived peptides and possible mechanisms of action.

Animal and Specie	Peptide	In Vitro Activity (IC_50_)	In Vivo Treatment	Effects and Mechanism of Action (When Investigated)	SI	References
Scorpions						
Scorpion *Leiurus quinquestriatus*	Chlorotoxin	300 nM Cl^−^ channel inhibition without death (D54-MG)	-	Blocks Cl^−^ channel and CLC-3.	-	[77]
Scorpion *Leiurus quinquestriatus*	M-CTX-Fc-L-Dox	0.17 μM (U251MG-P1)	10 mg/kg (tail injection)	In vitro: Promotes targeted cellular uptake and doxorubicin internalization via MMP-2 interaction. In vivo: Suppresses in vivo tumor growth.	-	[78]
Scorpion *Leiurus hebraeus*	Lqh-8/6	16.6 μM (U87), 0.84 μM (F98) and 4.14 μM (RG-2)	-	In vitro: Induces apoptosis. Ex vivo: Binds selectivety to F98 cell implanted in rats.	-	[79]
Scorpion *Androctonus australis* Hector	KAaH1	- (U87)	-	Inhibits cancer cell adhesion and migration.	-	[12]
Scorpion *Androctonus australis* Hector	KAaH2	7.96 μM (U87)	-	Blocks Kv1.1 and Kv1.3 and reduces EGFR, ERK, AKT and JNK proteins.	-	[12]
Jellyfishes						
Jellyfish *Pelagia noctiluca*	F1 fraction	125 μg/mL (U87)	-	Inhibits adhesion of U87 cells to fibrinogen (adhesion IC_50_ = 5 μg/mL) and inhibits cell proliferation.	-	[81]
Jellyfish *Pelagia noctiluca*	F3 fraction	179 μg/mL (U87)	-	Reduces cell viability and inhibits cell proliferation.	-	[81]
Spiders						
Spider *Phoneutria nigriventer*	SF3 and SF4	Active at 0.1 μg/mL (NG97)	-	Probably induces necrosis and enhances the antitumor effects of rapamycin in combination	-	[82]
Spider *Phoneutria nigriventer*	Phα1β and CTK 01512-2 (recombinant)	M059J, U-138M and U251-MG (0.3 at 100 pM)	GL261 murine (i.c.v.) 50 pmol/site	In vitro: Inhibits cell proliferation and induces apoptotic cell death. In vivo: Tumor size reduction		[83]
Spider *Lycosa erythrognata*	LyeTx I-b	29.20 μM (U87-MG) and 20.94 μM (U373-MG)	-	Induces necroptosis.	-	[84]
Snakes						
Snake *Cerastes cerastes*	Crude venom	0.12 μg/mL (SHSY5Y)	-	-	-	[85]
Snake *Cryptelytrops purpureomaculatus*	Crude venom	0.25 μg/mL (SHSY5Y)	-	-	-	[85]
Snakes *Naja anchietae* and *Naja senegalensis*	CTNanc1/CTNsen3	36.41 μg/mL (U87, U251 and U373)	-	Inhibited proliferation, induced apoptosis, altered cell cycle, and reduced migration	-	[86]
Other species						
Bee *Apis mellifera*	Melittin	0.274 μM (GAMG)	-	-	-	[80]
Snail *Conus virgo*	Crude venom	1.5 μg/mL (C6)	-	Induces apoptosis.	-	[74]

i.c.v.: intracerebro-ventricular injection; SI: selectivity index.

### 1.5. Colorectal Cancer

As with other types of cancer, the antitumor activity of toxins and bioactive peptides of animal origin is also being explored in colorectal cancer (CRC). The most studied venoms and/or peptides in CRC are from bees, scorpions, and snakes (Table 5). In vitro experiments have been conducted with various venoms from different animals, including bees [87], marine animals [88,89], snakes [90,91,92], and scorpions [93,94].

Regarding peptides from bee venom, melittin is the most extensively studied in colorectal cancer (CRC), demonstrating antitumor effects in both in vitro cell lines and in vivo models. One study showed melittin’s cytotoxicity against HCT116 cells (IC_50_ = 14.05 μg/mL) and suggested a synergistic interaction with phospholipase A_2_ (PLA_2_) from bee venom; however, no isobolographic analysis or mechanistic experiments were provided [95]. Another investigation reported 80% inhibition of HT-29 cells at 2 μM and reduction of HT-29 metastasis in Balb/c nude mice [96].

Melittin has demonstrated cytotoxicity against HCT116 cells (IC_50_ = 14.05 μg/mL), both in vitro and in vivo, through mechanisms involving cytolysis, pore formation, and induction of apoptosis [95,96,97]. In vitro studies on COLO205 and HCT-15 CRC cells revealed that melittin inhibits protein synthesis and translation. Within 1 min of treatment, membrane alterations including granulation, bleb formation, and cell swelling, were observed, followed by shrinkage and cell death at 15 min. Similar responses were observed in both cell lines, with IC_50_ values of 13 μg/mL for HCT15 and 11 μg/mL for COLO205 cells [98]. When conjugated to lactobionic acid–functionalized nanoparticles (LBA), melittin induced necrosis in HCT116 cells (IC_50_ < 10 μM), and an in vivo study using LBA–melittin at 2 mg/kg reported a significant reduction in tumor size in mice [99]. Modified variants of melittin have also been evaluated: D-melittin (with D-configured amino acids) exhibited IC_50_ values of 2.2 μM (free) and 11.6 μM (in micelles) against CT26 cells, and in vivo reduced tumor volume without improving survival, whereas D-melittin in micelles extended animal survival [97].

Despite its promising in vitro antitumor effects, systemic administration of melittin is significantly limited by severe side effects, including strong immune responses, hemolysis, thrombocytopenia, coagulation disorders, hepatotoxicity, and muscle damage [100,101,102,103]. To address these challenges, several studies have explored the incorporation of melittin into delivery systems—such as nanoparticles, chemical conjugates, and molecular modifications—to mitigate toxicity and enhance therapeutic efficacy [97,99,102,104]. One notable 2021 study demonstrated that replacing L-amino acids in melittin (L-melittin) with D-amino acids (D-melittin) markedly reduced the inflammatory response typically triggered by the native peptide. The resulting micellar nanoformulation (DMM) not only elicited a significantly milder immune reaction but also preserved melittin’s cytolytic activity against CT26 cells (IC_50_ = 11.6 µM) and induced pH-dependent hemolysis. Mechanistic investigations revealed that DMM promotes immunogenic cell death, indicating its potential role in antitumor immunotherapy. In vivo, DMM displayed a superior safety profile and more effective tumor growth inhibition in Balb/c mice compared to the free peptide. Although D-melittin alone did not improve survival relative to L-melittin, DMM-treated mice exhibited significantly smaller tumors and increased survival rates compared to those treated with PBS or the free peptide, supporting DMM’s potential as both a therapeutic agent and a targeted delivery system for antitumor peptides [97].

While bee peptides and venoms primarily exert their antitumor effects through cell membrane disruption and the induction of apoptosis or necrosis, scorpion venoms and their peptides appear to act mainly by promoting the generation of ROS and altering the cell cycle to induce apoptosis [93,94,105,106]. Recently, a novel peptide named Leptulipin was identified through in silico analyses of the venom of the scorpion *Hemiscorpius lepturus*. In vitro, Leptulipin exhibited antitumor activity against HT-29 colorectal cancer cells, with an IC_50_ value of approximately 40 μg/mL after 24 h of treatment. At 50 μg/mL, the peptide reduced cell proliferation by 20.13 ± 1.9%. Furthermore, treatment of HT-29 cells significantly increased the mRNA expression of the pro-apoptotic markers Bax and Casp9 while reducing the expression of the anti-apoptotic marker Bcl-2. Cell-cycle analysis also revealed an accumulation of cells in the G0/G1 phase accompanied by a reduction in the G2/M population [106].

Another peptide, Gonearrestide, described in 2018 and derived from the venom of *Androctonus mauritanicus* and *Androctonus australis*, exhibited dose-dependent antiproliferative activity against HCT116 CRC cells. Mechanistic investigations revealed that the peptide induced G1-phase cell-cycle arrest through inhibition of cyclin-dependent kinase 4 (CDK4) and upregulation of cyclin D3, p21, and p27. These findings were confirmed by transcriptomic and functional analyses. In nude mice, treatment with 50 or 100 μmol/L of Gonearrestide significantly reduced tumor growth, and immunohistochemical analyses further demonstrated reduced CDK4 expression in tumor tissues [105].

Recombinant actinoporin rHct-S3 from anemone *Heteractis crispa* have been demonstrated in vitro antitumor activity against HT-29 cell line with an IC^50^ of 7.3 µM as well as similar effects in non-tumor cells HEK-293 (8.5 µM) and JB6 Cl41 (8.6 µM). In functional assays, rHct-S3 inhibited EGF-induced neoplastic transformation in JB6 Cl41 cells (reductions of 10 ± 5%, 23 ± 2.5%, and 34 ± 0.2% at 1–4 µM) and reduced clonogenicity of HT-29 (25–47%), MDA-MB-231 (17–37%), and SK-MEL-28 (18–34%) within 1–4 µM, with performance comparable to the positive control cisplatin (3 µM) in some conditions. It exhibited anti-migratory effect in HT-29 cells (suppression of 33%, 50%, and 99% at 1, 2, and 4 µM, respectively), while the antiproliferative effect was moderate (≤31% at 96 h). Mechanistically, rHct-S3 suppressed MMP-2/MMP-9, activated/cleaved caspase-3 and PARP, and modulated Bax/Bcl-2, consistent with apoptosis and migration inhibition as key pillars of its antitumor activity [89].

Moreover, non-purified fractions derived from *Naja naja oxiana* venom were tested on primary cells isolated from human colorectal tissue (n = 20 patients), demonstrating marked selectivity toward tumor tissue when compared with adjacent normal tissue. Among these, Fraction 3 (F3) exhibited the highest cytotoxic effect, reducing cell viability with an IC_50_ of approximately 20 μg/mL in primary colorectal tumor cell cultures, while showing no significant toxicity in normal cells. Mechanistic analyses revealed that F3 activity was associated with increased production of reactive oxygen species (ROS), collapse of mitochondrial membrane potential, mitochondrial swelling, cytochrome c release, and subsequent caspase-3 activation, ultimately leading to apoptosis. A significant depletion of ATP levels was also observed in tumor cell mitochondria, further supporting the mitochondrial intrinsic pathway as the principal target of F3. Collectively, these findings suggest that *Naja naja oxiana* venom, particularly its F3 fraction, constitutes a promising candidate for the development of novel therapeutic strategies against human colorectal cancer [92].

The venom of the Ottoman viper *Montivipera xanthina* exhibits selective cytotoxic activity with potential therapeutic implications. In vitro assays demonstrated that crude venom significantly inhibited the proliferation of several human cancer cell lines, with IC_50_ values against HT-29 of 12.7 μg/mL (24 h) and 6.3 µg/mL (48 h). No cytotoxic effect was observed on Hep3B hepatoma or non-tumorigenic Vero kidney epithelial cells, indicating a degree of selectivity toward certain tumor types. Morphological changes such as cell rounding, detachment, and growth inhibition were consistent with venom-induced cytotoxicity. These results demonstrate that *M. xanthina* venom possesses anticancer properties, with higher sensitivity observed in hormone-dependent cancer lines supporting its potential as a source of bioactive molecules for anticancer drug development [90].

Moreover, snake venoms, obtained from *Bitis arietans*, *Cerastes gasperettii*, *Echis coloratus*, and *Echis pyramidum*, exhibit anticancer activity against human colorectal adenocarcinoma HCT-8 cell line. Treatment with venom (10 µg/mL) caused a dramatic reduction in colony formation (≈85–95% inhibition), suppressed cell motility (60–90% reduction), and inhibited Matrigel invasion (50–90%) compared to controls. Mechanistically, venom exposure significantly increased reactive oxygen species (ROS) production, correlating with elevated apoptosis rates (up to 70% apoptotic cells detected by Annexin V/PI staining). Furthermore, venom-treated cells displayed marked downregulation of the pro-inflammatory cytokines IL-6 and IL-8, both of which are associated with tumor progression [91]. Colorectal carcinoma (CRC) cells (RKO-AS-45-1) were successfully propagated in the CAM (chicken chorioallantoic membrane model) of colorectal cancer and LyeTxI-b (30 µM), a synthetic peptide derived from the natural peptide LyeTxI, purified from the venom of the spider *Lycosa erythrognatha*, induced arteriole occlusion and decreased vascularization similarly to cisplatine (34 µM), relative to controls. Using this model, it was possible to evaluate the efficacy of LyeTxI-b as an antitumor agent in CRC [107].

**Table 5:** Anti-colorectal-cancer activity of toxin-derived peptides and possible mechanisms of action.

Animal and Specie	Peptide	In Vitro Activity (IC_50_)	In Vivo Treatment	Effects and Mechanism of Action (When Investigated)	SI	References
Bees						
Bee *Apis mellifera syriaca*	Melittin, PLA2 and Melittin-PLA2 conjugated	Melittin 14.05 μg/mL PLA2: >50 μg/mL (HCT116)	-	Induces cancer cell cytotoxicity maily when isin combination (synergistic effect)	-	[95]
Bee *Apis mellifera*	Melittin	2 μM (HT-29)	1.5 mg/kg (i.t.)	Induces necrosis	-	[96]
Bee *Apis mellifera*	Melittin	11 μg/mL (COLO205) and 13 μg/mL (HCT-15)	-	Induces cell membrane damage (granulation, swelling and blebbing). Induces apoptosis.	-	[98]
Bee *Apis mellifera*	LBA-mellitin nanocomplex	1 μM (HCT116)	2 mg/kg (i.p.)	Increases the antitumor efficacy of melittin by targeting tumors and controlling intracellular release, leading to cell death through membrane pore formation.	-	[99]
Bee *Apis mellifera*	D-Melittin and DMM	2.2 μM (D-Melitin) and 11.6 μM (DMM) (CT26)	2 mg/kg of D-Melitin and 5 mg/kg of DMM (i.v.)	Decreases immune response maintaining cytolytic potential in vitro. In vivo, DMM was safety and inhibited tumor growth.	-	[97]
Scorpions						
*Scorpion Androctonus bicolor*, *Androctonus crassicauda*, and *Leiurus quinquestriatus*	Crude venom	>40 μg/mL (lHCT-8) and >60 μg/mL (HCT116)	-	Reduces tumor cell motility. Inhibits cell proliferation.	-	[94]
*Scorpion Androctonus bicolor*, *Androctonus crassicauda*, and *Leiurus quinquestriatus*	Crude venom encapsulated by liposomes	200 μg/mL (HCT-8)	-	Increases ROS levels and induces apoptosis.	-	[93]
Scorpion *Hemiscorpius lepturus*	Leptulipin	40 μg/mL (HT-29)	-	Inhibits cell proliferation; upregulates Bax and Casp9, downregulates Bcl-2, and induces G0/G1 cell-cycle arrest.	-	[106]
Scorpion *Androctonus mauritanicus* and *Androctonus australis*	Gonearrestide	~50 μm/L (HCT116)	50 and 100 μg/tumor) (p.t.)	In vitro: Induces G1-phase cell-cycle arrest through CDK4 inhibition and upregulation of cyclin D3, p21, and p27. In vivo: Suppresses tumor growth and reduces CDK4 expression in tumor tissues.	-	[105]
Snakes						
Snake *Naja naja oxiana*	Fraction 3	20 μg/mL (primary colon cancer cells)	-	Induces ROS-mediated apoptosis via mitochondria damage.	-	[92]
Snake *Montivipera xanthina*	Crude venom	12.7 μg/mL in 24 h and 6.3 μg/mL in 48 h (HT-29)	-	Exhibits cytotoxic activity.	-	[90]
Snakes *Bitis arietans*, *C. gasperettii*, *E. coloratus*, and *Echis pyramidum*	Crude venom (V1 *Bitis arietans*, V2 *C.gasperettii*)	10 μg/mL (HCT-8)	-	Increases ROS release. Reduces cell motility, cell invasion, colony formation. Decreases IL-6 and IL-8 cyto-kines and RhoC and p-Erk1/2. Induces apoptosis.	-	[91]
Other species						
Sea anemone *Heteractis crispa*	rHct-S3	6.8 μM (HT-29) and 8.5 μM (HEK-293)	-	Inhibits colony formation and cell migration. Induces apoptosis.	-	[89]
Spider *Lycosa erythrognatha*	LyeTxI-b	-	30 µM	Induces arteriole occlusion and reduces vascularization.	-	[107]

DMM: D-melittin micelle; i.p.: intraperitoneal; i.v.: intravenous; p.t.: peritumoral; ROS: reactive oxygen species; SI: selectivity index.

### 1.6. Ovary Cancer

Ovarian cancer remains one of the most lethal gynecological malignancies, largely due to its asymptomatic progression and frequent diagnosis at advanced stages. Novel therapeutic strategies, including toxin-derived peptides, have gained attention for their ability to selectively target cancer cells through diverse mechanisms [108]. Table 6 provides a comprehensive summary of the main findings regarding the anticancer activity of toxin-derived peptides and crude venoms in ovarian cancer, including key in vitro studies and their associated cellular pathways.

Conotoxins from cone snail venoms have demonstrated promising cytotoxic activity in ovarian cancer models. In vitro assays using ID8 ovarian cancer cells revealed that peptides Mr3e.1 (*Conus marmoreus*), Tx3a.1 (*Conus textile*), and Vi14b (*Conus virgo*) exerted dose-dependent cytotoxic effects, with IC_50_ values of 54.97 µM, 24.9 µM, and 116 µM, respectively [109]. Melittin, a cationic peptide derived from the venom of the honeybee *Apis mellifera*, effectively inhibited the proliferation of both cisplatin-sensitive (A2780) and cisplatin-resistant (A2780 CR) ovarian cancer cells, presenting IC_50_ values of 6.8 µg/µL and 4.5 µg/µL, respectively. Additionally, melittin treatment led to increased intracellular LDH release, suggesting membrane disruption as a key cytotoxic mechanism [110].

Marine-derived venom components have also shown potential against ovarian cancer. A protein cocktail isolated from the polychaete *Eulalia* sp. (including 14-3-3 protein, Hsp70, Rab3, arylsulfatase B, and serine protease) demonstrated cytotoxicity against A2780 cells with an IC_50_ of 0.08 µg/µL. Mechanistic studies indicated cell-cycle arrest at the G2/M phase and upregulation of caspase-8, indicating activation of the extrinsic apoptotic pathway. Signs of autophagic cell death were also observed, suggesting a dual mechanism of action [111].

Venom-derived compounds have demonstrated significant anticancer activity in ovarian cancer models by inhibiting cell proliferation, disrupting membrane integrity, inducing apoptosis, and promoting autophagy. Despite these promising results, ovarian cancer remains underexplored in venom-based research. Greater scientific investment is needed to fill this gap and fully realize the therapeutic potential of venom-derived peptides in ovarian cancer treatment.

### 1.7. Prostate Cancer

Prostate cancer is the second most frequently diagnosed cancer in men and a leading cause of cancer-related death globally. Although androgen deprivation therapy is initially effective, many patients progress to castration-resistant prostate cancer, which has limited treatment options [112]. In this context, toxin-derived peptides have gained attention for their selective cytotoxicity and ability to modulate signaling pathways involved in tumor progression and metastasis. Table 7 summarizes key findings on the anticancer activity of toxin-based peptides against prostate cancer, highlighting relevant in vitro and in vivo results.

Peptides AaHIV, AaHI, and AaHII, isolated from the venom of the scorpion *Androctonus australis*, have demonstrated cytotoxic effects on DU145 prostate cancer cells. Among the three peptides, AaHIV induced cytotoxicity at the lowest concentration, re-sulting in an IC_50_ value of 15 µM. In an in vivo toxicity assay, this peptide exhibited a median lethal dose (LD_50_) of 15 ng after intracerebroventricular (i.c.v.) administration in C57BL/6 mice [113].

Lycosin-I, a peptide toxin isolated from spider venom, also showed anticancer effects against prostate cancer cells. Treatment with 10 or 20 µM of lycosin-I led to marked morphological alterations and significantly inhibited the proliferation and migration of DU145 and PC-3 cells. Mechanistic studies indicated a dose-dependent increase in cleaved caspase-3 and caspase-9 levels, pointing to apoptosis induction. Furthermore, lycosin-I suppressed the STAT3 signaling pathway, which is often upregulated in prostate cancer, thereby inhibiting cell migration and promoting apoptosis. In vivo assays confirmed that lycosin-I reduced tumor growth in DU145 and PC-3 xenograft models in a dose-dependent manner. Notably, low concentrations were sufficient to suppress invasion and metastasis, while higher concentrations also induced apoptosis in tumor tissues [114].

Toxin-derived peptides have demonstrated promising potential as therapeutic agents against prostate cancer by suppressing tumor growth, inhibiting metastasis, and inducing apoptosis through key signaling pathways such as STAT3. These findings support further investigation into the development of peptide-based treatments tailored for prostate cancer.

### 1.8. Head and Neck Cancer

Head and neck cancers, particularly squamous cell carcinomas, remain a major clinical challenge due to their aggressive behavior and limited treatment responsiveness. Recent studies have highlighted the potential of toxin-derived peptides as selective anticancer agents in this context, capable of modulating apoptotic pathways and enhancing therapeutic responses [6,115]. Table 8 presents the principal findings regarding the anticancer effects of animal toxin peptides in head and neck cancers, including key in vitro and in vivo studies and their underlying cellular mechanisms.

Melittin, as previously cited in this text, a major component of bee venom from *Apis mellifera*, has demonstrated promising antitumor effects in head and neck cancers. In esophageal squamous cell carcinoma (ESCC) cell lines, melittin significantly enhanced the response of ECA109 and TE13 cells to ionizing radiation, with IC_50_ values of 1.88 μM and 1.64 μM, respectively. At concentrations of 0.5 and 1 μM, melittin reduced the post-irradiation survival fraction of ECA109 cells from 0.81 to 0.66 and 0.35, respectively, and of TE13 cells from 0.74 to 0.65 and 0.45, indicating increased sensitivity to radiotherapy. Furthermore, the combination of melittin and radiation enhanced apoptosis through Bax upregulation. These effects were confirmed in vivo using ECA109 tumor-bearing mice, where melittin treatment led to enhanced tumor regression and apoptotic activity [116].

Similarly, BmKn-2, a cationic antimicrobial peptide isolated from the scorpion *Mesobuthus martensii Karsch*, induced selective cytotoxicity in head and neck cancer cell lines. In HSC-4 and KB oral squamous carcinoma cells, BmKn-2 exhibited IC_50_ values of 26 μg/mL and 34 μg/mL, respectively. Treatment resulted in decreased BCL-2 expression and increased levels of BAX, cleaved caspase-3, and caspase-7, suggesting activation of the intrinsic apoptotic pathway. The upregulation of initiator caspase-9, with no effect on caspase-8, further supports this mechanism [117].

Pardaxin, a peptide derived from the marine fish *Pardachirus marmoratus*, demonstrated significant antiproliferative activity against oral squamous cell carcinoma (SCC-4) cells, with an IC_50_ below 10 μg/mL. After 24 h of treatment, pardaxin reduced cyclin B1 protein levels and upregulated p53 expression in a dose-dependent manner (5, 10, and 25 μg/mL). These molecular changes were associated with a reduction in the G0/G1 phase and arrest in the G2/M phase of the cell cycle, suggesting that pardaxin’s effects on cell growth may be mediated by modulation of cyclin B1 and p53. In vivo, pardaxin treatment significantly reduced carcinogenesis in the DMBA-induced hamster buccal pouch model, likely through the suppression of prostaglandin E2 levels [118].

Further supporting the therapeutic relevance of venom-derived molecules, rLj-RGD4, a recombinant peptide from the lamprey *Lampetra japonica* salivary gland, exhibited antitumor activity in a human laryngeal carcinoma (Hep-2) xenograft model. Containing four RGD motifs, rLj-RGD4 suppressed tumor growth in a dose-dependent manner (12.5–50.0 μg/kg), reduced angiogenesis by modulating VEGF and CD34 expression, and induced caspase-dependent apoptosis via the mitochondrial pathway. The peptide also inhibited endothelial cell migration and adhesion through regulation of the PI3K/AKT signaling axis, contributing to its angiogenic and antitumoral profile [119].

Despite these promising results, head and neck cancers are still underrepresented in venom-derived peptide research. Greater scientific investment is needed to expand therapeutic alternatives and translate these experimental findings into clinical applications.

**Table 8:** Anti-head–neck-cancer toxin-derived peptides, their activities, and possible mechanisms of action.

Animal and Specie	Peptide	In Vitro Activity (IC_50_)	In Vivo Treatment	Effects and Mechanism of Action (When Investigated)	SI	References
Fishes						
HanFish *Pardachirus marmoratus*	Pardaxin	<10 μg/mL (SCC 4)	25–75 mg/kg (topical administration to the oral mucosa)	In vitro: Increases caspase-3 and p53 protein level. Decreases B1 protein level. Arrest cells in G2/M phase. Induces apoptosis. In vivo: inhibits tumor growth.	-	[118]
Fish *Lampetra japonica*	rLj-RGD4	-	12.5–50.0 μg/kg (i.p.)	Inhibits tumor growth. Decreases P-FAK, P-PI3K, Bcl-2, P-Akt, and VEGF, and increases caspase-3, caspase-9, and Bax tumor tissue expression. Induces apoptosis.	-	[119]
Other species						
Bee *Apis mellifera*	Melittin	1.88 μΜ (ECA109) and 1.64 μΜ (TE13)	5 mg/kg (i.p.)	In vitro: increases Bax expression. Induces apoptosis. In vivo: Inhibits tumor growth	-	[116]
Scorpion *Mesobuthus martensii Karsch*	BmKn-2	26 μg/mL (HSC4) and 34 μg/mL (KB)	-	Induces morphological tumor cell alterations, includding nuclear disintegration and segregated bodies. Increases caspase-9 expression. Induces apoptosis.	3.84 (HGC/HSC4) 2.94 (HGC/KB)	[117]

i.p.: intraperitoneal; SI: selectivity index.

### 1.9. Leukemia

Leukemia, a group of hematologic malignancies affecting blood and bone marrow, continues to present therapeutic challenges, especially in resistant or relapsed cases. In this context, venom-derived peptides have emerged as promising anticancer agents due to their cytotoxic activity and diverse mechanisms of action [120]. Table 9 summarizes key findings on the anticancer activity of toxin-derived peptides against leukemia cells, highlighting in vitro efficacy and underlying molecular.

Melittin exerts cytotoxic effects against both acute lymphoblastic leukemia (CCRF-CEM) and chronic myelogenous leukemia (K-562) cell lines, with IC_50_ values of 3.6 µM and 1 µM, respectively. The peptide promotes apoptosis via mitochondrial membrane depolarization and induces more than 90% cell death within 24–48 h at 100 µM. Notably, melittin is non-toxic to peripheral blood mononuclear cells (PBMCs), suggesting preferential activity toward leukemia cells [121]. In U937 cells, synthetic melittin (1 µM) rapidly induced cell lysis within 10–15 min, with early morphological changes and increased intracellular and extracellular arachidonic acid levels, possibly due to acylhydrolase activity during membrane disruption [122].

Latarcin 2a, a short peptide derived from the venom of the spider *Lachesana tarabaevi*, exhibits selective in vitro cytotoxicity against the K562 chronic myelogenous leukemia cell line, with an EC_50_ of 3.3 μM. In contrast, it shows significantly lower toxicity toward normal blood leukocytes, with an EC_50_ of 19.5 μM, indicating preferential action against leukemic cells. The cytotoxic effect of Latarcin 2a is initiated by the formation of membrane pores approximately 3.7 nm in diameter, which display higher permeability to anionic molecules than to cationic ones. This pore formation leads to membrane blebbing and cellular swelling, ultimately resulting in cell death. Additionally, Latarcin 2a undergoes self-facilitated internalization, accumulates within mitochondria, and disrupts mitochondrial function. Despite inducing the externalization of phosphatidylserine (PS), a marker commonly associated with apoptosis, Latarcin 2a does not activate apoptotic pathways, suggesting a distinct, apoptosis-independent mechanism of cell death [120]. Another venom-derived peptide from *Lachesana tarabaevi*, Latarcin 3a, and its synthetic analogue Lt-MAP2, have been shown to exert antitumor activity across several leukemia cell lines. Latarcin 3a inhibited the growth of C1498 and K562 leukemia cells with IC_50_ values of 76.9 µg/mL and 78.2 µg/mL, respectively. Lt-MAP2 demonstrated greater activity, with IC_50_ values of 43.9 µg/mL for C1498 and 44.0 µg/mL for K562 cells, highlighting the potential for structural optimization to enhance therapeutic efficacy [123].

Another peptide derived from spider venom is Lycosin-I, a molecule isolated from *Lycosa singorensis*, which exhibits anticancer activity across several leukemia cell lines, including U937, HL-60, MV4:11, and K562, with IC_50_ values ranging from 1.66 to 4.2 μM. In K562 cells, Lycosin-I induces apoptosis, G1-phase cell cycle arrest, and ferroptosis, mechanisms mediated by increased intracellular Fe^2+^ levels and elevated ROS formation. These effects are associated with suppression of the PI3K-AKT-mTOR signaling pathway and activation of autophagy at low concentrations. In vivo, intraperitoneal administration of Lycosin-I significantly inhibited K562 tumor growth in nude mouse xenograft models without inducing observable side effects, reinforcing its therapeutic potential for leukemia treatment [35].

In a similar vein, ICD-85, a peptide complex composed of three bioactive peptides extracted from the venoms of the Iranian brown snake (*Gloydius halys*) and the yellow scorpion (*Hemiscorpius lepturus*), significantly suppressed HL-60 promyelocytic leukemia cell proliferation. With a reported IC_50_ of 0.04 ± 0.015 µg/mL, ICD-85 treatment induced hallmark features of apoptosis, including nuclear condensation, mitochondrial swelling or degradation, dilation of the endoplasmic reticulum, increased cytoplasmic vacuolization, and DNA fragmentation [124].

Another class of venom components with potential anticancer effects are phospholipases A2 (PLA2s), which have been reported to exhibit both cytotoxic and therapeutic effects against leukemia cells [125]. A notable example is BthTX-I, a basic myotoxic PLA2 isolated from the venom of the snake *Bothrops jararacussu*. This toxin induced significant cytotoxicity in HL-60 leukemia cells with an IC_50_ ranging from 5 to 10 μg/mL, primarily through the induction of both apoptotic and necrotic cell death pathways [126].

Further evidence of the antileukemic potential of venom components comes from crude venom extracted from the spider *Macrothele raveni*. This venom exhibited dose- and time-dependent growth inhibition in K562 leukemia cells, with an IC_50_ of 5.1 µg/mL, while showing lower toxicity to normal human lymphocytes (IC_50_ ≈ 36.4 µg/mL), suggesting a degree of selectivity toward malignant cells. Morphological analyses of venom-treated K562 cells revealed classic apoptotic changes such as chromatin condensation and DNA fragmentation. These effects were supported by molecular findings showing activation of caspase-3 and caspase-8, as well as PARP cleavage, indicating activation of intrinsic and extrinsic apoptotic pathways [127].

Melittin, BthTX-I, Latarcins, Lycosin-I, and other venom-derived peptides represent promising candidates for leukemia therapy, exhibiting diverse mechanisms of action and significant antileukemic activity. Although most evidence remains limited to preclinical studies, some compounds have demonstrated preferential activity toward malignant cells and encouraging in vivo efficacy. Continued experimental and clinical investigations are required to establish their safety profiles and therapeutic potential for future leukemia treatment strategies.

**Table 9:** Anti-leukemia toxin-derived peptides, their activities, and possible mechanisms of action.

Animal and Specie	Peptide	In Vitro Activity (IC_50_/EC_50_)	In Vivo Treatment	Effects and Mechanism of Action (When Investigated)	SI	References
Bees						
Bee *Apis mellifera*	Melittin	3.6 μM (CCR-CEM) and 1 μM (K-562)	-	Decreases in mitochondrial membrane potential. Increases caspases 3/7 pathway activation. Induces apoptosis.	-	[121]
Bee *Apis mellifera*	Synthetic Melittin	- (U937)	-	Increases in total arachidonic acid levels. Induces pore formation and membrane disruption.	-	[122]
Spiders						
Spider *Lachesana tarabaevi*	Latarcin 2a	3.3 μΜ (K562)	-	Induces morphological tumor cell alterations, including membrane blebbing and swelling and plasmatic membrene pores.	5.9	[120]
Spider *Lachesana tarabaevi*	Latarcin 3a	76.9 µg/mL (C1498) and 78.2 µg/mL (K562)	-	Exhibits cytotoxic activity.	-	[123]
Spider *Lachesana tarabaevi*	Lt-MAP2	43.9 µg/mL (C1498) and 44.0 µg/mL (K562)	-	Exhibits cytotoxic activity.	-	[123]
Spider *Lycosa singorensis*	Lycosin-I	Approximately 2 µM (K562)	9 mg kg^−1^ D-Lycosin-I (i.p.)	In vitro: Induces apoptosis, ferroptosis, and G1-phase cell cycle. Increases intracellular Fe^2+^ and ROS levels. Suppresses the PI3K-AKT-mTOR signaling pathway and activates autophagy. In vivo: Inhibits tumor growth in K562 xenograft models without observable side effects.	-	[35]
*Spider Macrothele raveni*	Crude venom	5.1 μg/mL	-	Induces morphological tumor cell alterations, including nuclei condensation and DNA fragmentation. Increases caspases 3 and 8 activation. Induces apoptosis.	-	[127].
Other species						
Mix between peptides from snake *Agkistrodon halys* scorpion *Hemiscorpius lepturus*	ICD-85	0.04 ± 0.015 μg/mL (HL60)	-	Induces morphological tumor cell alterations, including nuclear material condensation, endoplasmic reticulum dilatation, mitochondrial swelling or degradation increase in cytoplasmic vacuolization, reduction or loss of cytoplasmic projections, and DNA fragmentation.	-	[124]
Snake *Bothrops jararacussu*	BthTX-I	Between 5 and 10 μg/mL	-	Induces apoptosis and necrosis.	-	[126]

For comparative purposes, IC_50_ values or equivalent cytotoxicity parameters reported in the original studies (EC_50_) are presented when available. i.p.: intraperitoneal; ROS: reactive oxygen species; SI: selectivity index.

### 1.10. Liver Cancer

Hepatocellular carcinoma (HCC) is the most common form of primary liver cancer and ranks among the leading causes of cancer-related deaths worldwide. Its development is often associated with chronic infections by hepatitis B and C viruses, as well as exposure to aflatoxins, alcohol abuse, and metabolic syndromes. Despite the availability of potentially curative options, such as surgical resection, liver transplantation, and ablative therapies, many patients present with advanced-stage disease or develop recurrence, necessitating the development of complementary or alternative treatments. Among emerging strategies, toxin-derived peptides have garnered interest due to their capacity to interfere with tumor growth and metastasis through specific molecular targets. Although research on the use of toxin peptides in liver cancer remains limited, promising preliminary data are beginning to surface. Table 10 compiles key findings on toxin-derived peptides studied in liver cancer models, with an emphasis on their in vitro and in vivo antitumor effects and associated molecular mechanisms.

One of the most studied venom peptides in this context is melittin, previously cited with relevant activity in other cancer types. Melittin has demonstrated significant inhibitory effects on the aggressive HCC cell line MHCC97H, which is characterized by elevated Rac1 activity, an important mediator of cytoskeletal remodeling, cell motility, and metastasis. In vitro, melittin reduced MHCC97H cell viability and migration with an IC_50_ of 4.06 µg/mL, correlating with suppressed Rac1 activity and cytoskeletal depolymerization. Mechanistically, melittin inhibited MKK4, JNK, and c-Jun signaling in a dose-dependent manner. In vivo, administration of melittin at 80 μg/kg/day significantly inhibited tumor growth and metastasis in nude mouse models, confirming its Rac1-dependent anti-metastatic activity [128].

In another study, Moroccan scorpion venom, from *Buthus occitanus*, and its seven chromatographic fractions were evaluated for hepatotoxicity and antitumor potential. At 10 µg/mL, all fractions showed low toxicity toward Fa2N-4 normal hepatocyte cells, maintaining cell viability above 80%. In contrast, the same concentration of fraction F3 significantly reduced the surface area of 3D heterotypic spheroids composed of Huh7.5 liver cancer cells by 78.91%, indicating selective antitumor activity and disruption of tumor architecture [129]. Similarly, Moroccan cobra (*Naja haje*) venom and its fractions were tested against this same preparation, 3D spheroids of Huh7.5. While fraction F5 showed only minor effects at 10 µg/mL, higher concentrations (50 µg/mL) of fractions F1, F3, and F13 moderately reduced the spheroid area. Fractions F4, F5, F7, F8, and the crude venom led to significant suppression of spheroid proliferation, ultimately decreasing their size, thus suggesting a broader cytostatic and possibly cytotoxic effect on liver cancer spheroids [130].

The venom of the scorpion *Odontobuthus bidentatus* yielded three fractions with cytotoxic activity against HepG2 cells in an in vitro 3D model, with fractions 5, 6, and 10 exhibiting IC_50_ values of 1.291, 1.327, and 1.356 μg/mL, respectively. These fractions exert their effects by inducing oxidative stress, as evidenced by the data presented in the study, along with increased caspase-3 activity, enhanced cytochrome C release, and direct confirmation of apoptosis through comet assay analysis [131]. In summary, although still in early stages, the application of toxin-derived peptides in hepatocellular carcinoma models reveals a promising therapeutic direction, particularly in cases characterized by high metastatic potential. Melittin has shown robust and mechanistically defined anti-HCC activity in both, in vitro and in vivo systems, while scorpion and snake venom fractions demonstrate selective cytotoxicity against 3D liver tumor spheroids with minimal effects on healthy hepatocytes.

**Table 10:** Anti-liver-cancer toxin peptides, their activities, and possible mechanisms of action.

Animal and Specie	Peptide	In Vitro Activity (IC_50_)	In Vivo Treatment	Effects and Mechanism of Action (When Investigated)	SI	References
Scorpions						
Scorpion *Buthus occitanus*	F3 fraction	- (Huh7.5)	-	Exhibits cytotoxic activity. Decreases spheroid size.	-	[129]
Scorpion *Odontobuthus bidentatus*	F5	1.291 μg/mL (HepG2 3D model)	-	Induces apoptosis and oxidative stress.	-	[131]
Scorpion *Odontobuthus bidentatus*	F6	1.327 μg/mL (HepG2 3D model)	-	Induces apoptosis and oxidative stress.	-	[131]
Scorpion *Odontobuthus bidentatus*	F10	1.356 μg/mL (HepG2 3D model)	-	Induces apoptosis and oxidative stress.	-	[131]
Other species						
Bee *Apis mellifera*	Melittin	4.06 μg/mL (MHCC97H)	80 μg/kg/day (i.v.)	In vitro: inhibits the viability and migration. Supress Rac1 activity and tumor cell microfilament depolymerization In vivo: Inhibits tumor growth and metastasis.	-	[128]
Cobra *Naja haje*	Venom fractions	- (Huh7.5)	-	Exhibits cytotoxic activity.	-	[130]

i.v.: intravenous; SI: selectivity index.

## 2. Conclusions

Peptides have shown interesting activities in the different models of diseases in vitro and in vivo. Specifically, all data in the literature point out the anticancer peptides being promising as good leads compounds and candidates to develop new bio-therapeutics to treat the different types of cancer. Some groups are widely studied, such as snakes, scorpions, and bees. It is notable that melittin, from bee *Apis mellifera*, has been tested in many cancer cells models, being a good example of a multiactivity peptide, with investigations even in different cancers, as well as against bacteria and different viruses. However, the relative high toxicity of melittin to the organisms must be considered. Chemical modification or formulation of this molecule are some strategies to try to reduce such toxicity. The known amino acid sequences of the main bioactive peptides exhibiting anticancer activity in the cancer types discussed in this review are presented in Table 11. Despite the promising anticancer activity reported for animal venom-derived peptides, several limitations of the current evidence should be considered. Most studies remain restricted to in vitro experiments, with comparatively few investigations progressing to in vivo validation. In addition, many studies evaluated only a limited number of cancer cell lines and frequently lacked toxicity assessments in non-tumoral cells, making it difficult to establish selectivity profiles and therapeutic windows and the calculation of selectivity indices, which are important parameters for comparing the translational potential of different compounds. Comparisons among studies are further complicated by differences in peptide origin, purification strategies, structural modifications, delivery systems, experimental conditions, and biological endpoints. Moreover, although multiple molecular targets and signaling pathways have been implicated in the antitumor effects of these compounds, mechanistic evidence remains incomplete for a substantial proportion of the peptides investigated.

Another relevant aspect is the unequal distribution of evidence across cancer types. A considerable proportion of the studies identified focused on breast cancer models, whereas highly prevalent and aggressive malignancies, including pancreatic, gastric, skin, and certain brain cancers, remain underrepresented. Furthermore, information regarding pharmacokinetics, biodistribution, systemic toxicity, and long-term safety remains scarce for most venom-derived peptides. Expanding the investigation of venom-derived peptides to these understudied malignancies, together with a more comprehensive characterization of their molecular targets, mechanisms of action, selectivity, and safety profiles, will be essential for advancing the preclinical development of these compounds. Collectively, these efforts may contribute to the identification of candidates with greater translational potential and support their progression toward clinical evaluation. Nevertheless, the structural diversity and broad spectrum of biological activities exhibited by animal venom-derived peptides continue to highlight their relevance as a source of novel anticancer agents.

## Figures and Tables

**Table 6:** Anti-ovary-cancer activity of toxin-derived peptides and possible mechanisms of action.

Animal and Specie	Peptide	In Vitro Activity (IC_50_)	In Vivo Treatment	Effects and Mechanism of Action (When Investigated)	SI	References
Snails						
Snail *Conus marmoreus*	Mr3.8	54.97 μM (ID8)	-	Exhibits cytotoxic activity.	-	[109]
Snail *Conus texile*	Tx3a.1	24.9 μM (ID8)	-	Exhibits cytotoxic activity.	-	[109]
Snail *Conus virgo*	Vi14b	116 μM (ID8)	-	Exhibits cytotoxic activity.	-	[109]
Other species						
Bee *Apis mellifera*	Melittin fusion with IL-2	4.5 μg/mL (A2780CR) and 6.8 μg/mL (A2780)	-	Induces necrosis.	-	[110]
Worm *Eulalia* sp.	14-3-3 protein, Hsp70, Rab3, Arylsulfatase B and serine protease	0.08 µg/uL^−1^ (A2780)	-	Induces apoptosis and autophagic cell death.	-	[111]

CR: cisplatin-resistant; SI: selectivity index;.

**Table 7:** Anti-prostate-cancer activity of toxin-derived peptides and possible mechanisms of action.

Animal and Specie	Peptide	In Vitro Activity (IC_50_)	In Vivo Treatment	Effects and Mechanism of Action (When Investigated)	SI	References
Scorpions						
Scorpion *Androctonus australis*	AaHIV	15 μM (DU145)	-	In vitro: Exerts cytotoxic effects on tumor cells. Activates Nav1.6 channels.	-	[113]
Scorpion *Androctonus australis*	AaHI	>15 μM (DU145)	-	Exerts low cytotoxic effects on tumor cells	-	[113]
Scorpion *Androctonus australis*	AaHII	>15 μM (DU145)	-	Exerts low cytotoxic effects on tumor cells	-	[113]
Other species						
Spider *Lycosa singoriensis*	Lycosin-I	>20 μM (DU145 and PC-3)	50 μg, 100 μg, and 200 μg (i.t.)	In vitro: Inhibits proliferation and migration. Activates caspases 3 and 9. Induces apoptosis. In vivo: inhibits the migration, invasion and metastasis. Inactivates STAT3 signaling pathway. Induces apoptosis.	-	[114]

i.t.: intratumoral; LD_50_: lethal dose 50%; SI: selectivity index.

**Table 11:** Toxin-derived peptides and their respective sequences.

Peptides	Sequences	Molecular Weight (Da)	References
Melittin (*Apis mellifera*)	GIGAVLKVLTTGLPALISWIKRKRQQ (maturated sequence)	2844.74 ‡	Uniprot: P01501
Melittin (*Apis cerana*)	MKFLVNVALVFMVVYISFIYAAPEPEPAPEAEAEADAEADPEAGIGAVLKVLTTGLPALISWIKRKRQQG (imature sequence)	8509.52 ‡	Uniprot: Q8LW54
Cytotoxin-II	LKCKKLVPLFSKTCPAGKNLCYKMFMVAAPHVPVKRGCIDVCPKSSLLVKYVCCNTDKCN	6631.40	Uniprot: P01441
NN-32	LKCNKLVPLF (first 10 residues)	~6700	[19]
AGAP linked by N-terminal to ATF peptide	VRDGYIADDKNCAYFCCRNAYCDDECKKNGAESGYCQWAGVYGNACWCYKLPDKVPIRVPGKCNGG	7322.20	[17]
Mastoparan	INLKALAALAKKIL	1478.99	[38]
Latroeggtoxin-V	SQAGEWGSGAGKGGGSGGSVRDAGGSFGKMEAAREEEYFRKQQKEQLKALKSHLHEEIDHHEEEIERLQEEIKRHKKKISDLAKEEKKIDG	10,166.12	[36]
Chlorotoxin	MCMPCFTTDHQMARKCDDCCGGKGRGKCYGPQCLCR	4005	Uniprot: P45639
Rusvikunin	HDRPTFCNLAPESGR	6936.89	[21]
Rusvikunin-II	HDRPTFCNLFPESGR (first 15 residues)	7108.36	[21]
LyeTxI-b	IWLTALKFLGKNLGKLAKQQLAKL	2739.36	[34]
LVTX-8	IWLTALKFLGKNLGKHLAKQQLSKL	2847.99	[60]
Apamin	MISMLRCIYLFLSVILITSYFVTPVMPCNCKAPETALCARRCQQHG (imature sequence)	5219.59 ‡	Uniprot: P01500
KAaH1	MMKLMLFSIIVILFSLIGSIHGADVPGNYPLDSSDDTYLCAPLGENPFCIKICRKHGVKYGYCYAFQCWCEYLEDKNVKI	9098.44 ‡	Uniprot: Q4LCT0
KAaH2	MMKLMLFSIIVILFSLIGSIHGADVPGNYPLDSSDDTYLCAPLGENPSCIQICRKHGVKYGYCYAFQCWCEYLEDKNVKI	9038.36 ‡	Uniprot: Q4LCS9
Lachesicidin	RhoB—KRFKKFFKK	1682.2	[20]
AaTs-1	IWKS	532.30 ‡	Uniprot: C0HLZ5
Promelittin	EPEAEADAEAGP (pro matured sequence)	1184.48 ‡	Uniprot: P01501
BmKn-2	FIGAIARLLSKIF	1448.81	[132]
Vincrostatin (VCN)	DAPANPCCDAATCKLTTGSQCADGLCCDQCKFMKEGTVCRRARGDDLDDYCNGISAGCPRNPHKGPATY	7257.10	[23]
ε-Ahx-TsAP-1	e-Amino-hexanoic—FLSLIPSLVGGSISAFK	1734.99	[13]
BmK AGAP	VRDGYIADDKNCAYFCGRNAYCDDECKKNGAESGYCQWAGVYGNACWCYKLPDKVPIRVPGKCNGG	7276.22	[16]
Antagonist of bombesin: Leu13ψ(CH2NH)Leu14-BN(6–14)	Gln-Trp-Ala-Val-Gly-His-Leu-ψCH2NH-Leu-NH2	~950	[133]
Lqh6	VRDGYIAQPENCVYHCIPDCDTLCKDNGGTGGHCGFKLGHGIACWCNALPDNVGIIVDGVKCHK	6798.10 ‡	Uniprot: P59356
Lycosin-I	RKGWFKAMKSIAKFIAKEKLKEHL	2886.68 ‡	Uniprot: P0DV70
Pardaxin	GFFALIPKIISSPLFKTLLSAVGSALSSSGGQE	3321.82	[118]
rLj-RGD4	AICHKQNYPMGTETQGDTRGDTRGDGDTRGARGDARRHGHNKHLHRMSAAVSECV	6401 ‡	[119]
Mr3.8	CCHWNWCDHLCSCCGS	1855.58	[109]
Tx3a.1	CCSWDVCDHPSCTCCG	1717.51	[109]
AaHIV	GRDGYIVDSKNCVYHCYPPCDGLCKKNGAKSGSCGFLVPSGLACWCNDLPENVPIKDPSDDCHK (mature sequence)	6887.09 ‡	Uniprot: P45658
AaHI	MNYLVMISLALLLMIGVESKRDGYIVYPNNCVYHCVPPCDGLCKKNGGSSGSCSFLVPSGLACWCKDLPDNVPIKDTSRKCTR	9054.39 ‡	Uniprot: P01479
AaHII	MNYLVMISLALLFVTGVESVKDGYIVDDVNCTYFCGRNAYCNEECTKLKGESGYCQWASPYGNACYCYKLPDHVRTKGPGRCHGR	7247.19 ‡	Uniprot: P01484
Dermaseptin-PP	ALWKDMLKGIGKLAGKAALGAVKTLV	2651.59	[61]
s-cal14.1a	Unknown mass	Unknown sequence	_
Leptulipin	DHDHCDGILSGETKCEEALDNCFK(an fragment)	12.81 KDa	[106]
TistH	ADMDFTGIAESIIKKIKETNAKPPA	2688.28	[50]
Triflavin	GEECD	~7500	[53]
Lqh-8/6	RCSPCFTTDQQMTKKCYDCCGGKGKGKCYGPQCICAPY	4170.75 ‡	Uniprot: P55966
Latarcin 2a	GLFGKLIKKFGRKAISYAVKKARGKH	2900.77 ‡	Uniprot: Q1ELU1
Latarcin3a	SWKSMAKKLKEYMEKLKQRA	2483.3	[123]
Lt-MAP1	LAKKLKEYLEKLV	1575.2	[123]
Lt-MAP2	LIKKLKEYLKKLI	1630.2	[123]
PhylloseptinPTa	FLSLIPKIAGGIAALAKHL	1931.19	[62]
PhylloseptinPha	FLSLIPAAISAVSALANHF	1940.07	[62]

‡: these masses were obtained by expasy program (https://web.expasy.org/compute_pi/ accessed on 16 December 2023) based on sequence from uniport (https://www.uniprot.org/ accessed on 16 December 2023) as in the references.

## Data Availability

No new data were created or analyzed in this study.
